# Relative contribution of type 1 and type 2 diabetes loci to the genetic etiology of adult-onset, non-insulin-requiring autoimmune diabetes

**DOI:** 10.1186/s12916-017-0846-0

**Published:** 2017-04-25

**Authors:** Rajashree Mishra, Alessandra Chesi, Diana L. Cousminer, Mohammad I. Hawa, Jonathan P. Bradfield, Kenyaita M. Hodge, Vanessa C. Guy, Hakon Hakonarson, Bone Mineral Density in Childhood Study, Heidi J. Kalkwarf, Joan M. Lappe, Vicente Gilsanz, Sharon E. Oberfield, John A. Shepherd, Andrea Kelly, Babette S. Zemel, Didac Mauricio, Nanette C. Schloot, Knud B. Yderstræde, Benjamin F. Voight, Stanley Schwartz, Bernhard O. Boehm, Richard David Leslie, Struan F. A. Grant

**Affiliations:** 1 Division of Human Genetics, The Children’s Hospital of Philadelphia, Philadelphia, PA USA; 2 Department of Genetics, Perelman School of Medicine, University of Pennsylvania, Philadelphia, PA USA; 3 Department of Immunobiology, Barts and the London School of Medicine and Dentistry, Queen Mary University of London, London, UK; 4 Center for Applied Genomics, The Children’s Hospital of Philadelphia, Philadelphia, PA USA; 5 Department of Pediatrics, Perelman School of Medicine, University of Pennsylvania, Philadelphia, PA USA; 6 Hospital Universitari Germans Trias i Pujol, Barcelona, Spain; 7 German Diabetes Center, Düsseldorf, Germany; 8 Odense University Hospital, Odense, Denmark; 9 Department of Systems Pharmacology and Translational Therapeutics, Perelman School of Medicine, University of Pennsylvania, Philadelphia, PA USA; 10 Main Line Health System, Wynnewood, PA USA; 11 Department of Internal Medicine I, Ulm University Medical Centre, Ulm, Germany; 12 LKC School of Medicine, Nanyang Technological University, Singapore and Imperial College, London, UK; 13 Divisions of Human Genetics and Endocrinology, The Children’s Hospital of Philadelphia, 3615 Civic Center Boulevard, Room 1102D, Philadelphia, PA 19104 USA; 14 Department of Immunobiology, Blizard Institute, 4 Newark Street, London, E1 2AT UK

**Keywords:** Latent autoimmune diabetes in adults, Genetic risk scores

## Abstract

**Background:**

In adulthood, autoimmune diabetes can present as non-insulin-requiring diabetes, termed as ‘latent autoimmune diabetes in adults’ (LADA). In this study, we investigated established type 1 diabetes (T1D) and type 2 diabetes (T2D) genetic loci in a large cohort of LADA cases to assess where LADA is situated relative to these two well-characterized, classic forms of diabetes.

**Methods:**

We tested the association of T1D and T2D GWAS-implicated loci in 978 LADA cases and 1057 non-diabetic controls of European ancestry using a linear mixed model. We then compared the associations of T1D and T2D loci between LADA and T1D and T2D cases, respectively. We quantified the difference in genetic risk between each given disease at each locus, and also calculated genetic risk scores to quantify how genetic liability to T1D and T2D distinguished LADA cases from controls.

**Results:**

Overall, our results showed that LADA is genetically more similar to T1D, with the exception of an association at the T2D *HNF1A* locus. Several T1D loci were associated with LADA, including the major histocompatibility complex region, as well as at *PTPN22*, *SH2B3*, and *INS*. Contrary to previous studies, the key T2D risk allele at *TCF7L2* (rs7903146-T) had a significantly lower frequency in LADA cases, suggesting that this locus does not play a role in LADA etiology. When constrained on antibody status, the similarity between LADA and T1D became more apparent; however, the *HNF1A* and *TCF7L2* observations persisted.

**Conclusion:**

LADA is genetically closer to T1D than T2D, although the genetic load of T1D risk alleles is less than childhood-onset T1D, particularly at the major histocompatibility complex region, potentially accounting for the later disease onset. Our results show that the genetic spectrum of T1D extends into adult-onset diabetes, where it can clinically masquerade as T2D. Furthermore, T2D genetic risk plays a small role in LADA, with a degree of evidence for the *HNF1A* locus, highlighting the potential for genetic risk scores to contribute towards defining diabetes subtypes.

**Electronic supplementary material:**

The online version of this article (doi:10.1186/s12916-017-0846-0) contains supplementary material, which is available to authorized users.

## Background

Diabetes is a heterogeneous group of diseases resulting in hyperglycemia due to insulin secretory dysfunction as well as insulin resistance. A substantial proportion of type 1 diabetes (T1D) cases present in adulthood, and despite the presence of diabetes-associated autoantibodies, the majority of these patients do not initially require insulin [1, 2]. The manifestation of this ‘latent autoimmune diabetes in adulthood’ (LADA) is clinically defined by (1) an adult age of onset, (2) at least one diabetes-associated autoantibody, and (3) the lack of requisite insulin treatment for at least 6 months after diagnosis. This definition overall represents approximately 5–10% of all cases of adult-onset diabetes, potentially the most frequent form of autoimmune diabetes [3, 4].

However, classifying adult-onset autoimmune T1D, including LADA, remains challenging. The need for insulin treatment is a clinical decision, while diabetes-associated autoantibodies are neither pathogenic nor categorical features of LADA. Decisions are further confounded by false positives when large numbers of patients are screened [5]. Since LADA has intermediate features between T1D and type 2 diabetes (T2D), there are limits to the current classification of diabetes. New paradigms are needed to distinguish LADA and ensure appropriate disease treatment and management.

Recently, several studies have used genetic information derived from diabetes-associated risk variants across the genome to reclassify diabetes [6]. To date, comprehensive genetic studies of T1D and T2D have uncovered dozens of distinct susceptibility loci for each of these two diseases [7–9]. Initial analyses of T1D loci in relatively small LADA cohorts have consistently shown an association with the T1D locus *HLA-DQB1*, which resides in the major histocompatibility complex (MHC) [3, 10, 11], as well as at *PTPN22* and *INS* [12, 13]. Similar analyses of T2D loci have suggested an association in LADA with the strongest T2D locus harboring *TCF7L2* [12, 14, 15] and the *ZMIZ1* locus [16]. A significant challenge of these studies has been the lack of statistical power due to the small number of LADA patients included. Thus, the genetic etiology of LADA remains largely unresolved.

To quantify the genetic liability to LADA contributed by genetic risk factors for T1D and T2D, we amassed the largest LADA cohort to date. By assessing the association of these variants in LADA, our objective was to place LADA along the etiological diabetes spectrum and reshape our understanding of the relationship between LADA and classic diabetes phenotypes.

## Methods

### Study populations and antibody testing

We ascertained 978 LADA cases from two studies, a European Union-funded multicenter study (Action LADA) and a German Research Council study (DFG: SFB 518, A1), each of which aimed to identify features of adult-onset autoimmune diabetes. A description of the participants and study design has been published elsewhere [2]. For this particular study, the criteria for LADA diagnosis was more strict to avoid potential false positives. All participants were diagnosed with LADA if they were aged 30–70 years, tested positive for diabetes-associated glutamic acid decarboxylase autoantibodies (GADA), and were not given insulin treatment for at least 6 months after diagnosis. Samples were tested for serum autoantibodies to GADA and insulinoma-associated antigen-2 (IA2A) (Additional file 1: Supplemental Methods, which also includes all genotyping methods and quality control).

The population-based control cohort comprised 1057 non-diabetic children of European ancestry, aged 5–20 years, enrolled in the Bone Mineral Density in Childhood Study (BMDCS). Subjects were randomly recruited from five different centers in the USA. As previously reported [17], enrollment criteria included healthy, normally developing children. Each participating center received approval of the study by their respective institutional review boards.

Since BMDCS consists of European-descent children ascertained from the USA, while the LADA cases were adults ascertained from the UK and Germany, we also leveraged 2820 healthy adult British birth cohort controls from the Wellcome Trust Case Control Consortium (WTCCC) [8] to act as an extra set of controls to verify our observations. Principal component analysis (PCA) showed that BMDCS controls were well-matched with cases despite ascertainment in the USA, while the WTCCC controls were stratified (Additional file 1: Figure S1) principally due to differences in the genotyping arrays used. Thus, BMDCS was used in the primary analyses, with verification in the WTCCC cohort. Our study also utilized publicly available childhood-onset T1D (n = 2000) and adult-onset T2D (n = 1999) Affymetrix 500 K genotype data from the WTCCC; these individuals were recruited within England, Scotland, and Wales [8]. Individual data from WTCCC is available through the Consortium’s Data access committee (http://www.wtccc.org.uk). The genomic inflation factor for the pruned genome-wide SNPs is 0.966 and the QQ-plot can be found in Additional file 1: Figure S2.

### Individual candidate SNP association tests

To investigate the role of previously discovered T1D and T2D variants in LADA, we tested 67 T1D SNPs (from Immunobase; http://www.immunobase.org, and 71 T2D SNPs from the T2D study led by the DIAbetes Genetics Replication And Meta-analysis (DIAGRAM) Consortium [9]). Association between each SNP and case/control status was assessed using a univariate linear mixed model within GEMMA [18]. This model accounts for population stratification and relatedness using the Wald test and the restricted maximum likelihood estimate of β. We tested each SNP in LADA cases versus BMDCS controls and in LADA cases versus T1D or T2D cases. Significant associations were called after Bonferroni correction for multiple testing. Analysis was performed for all LADA cases (n = 978), LADA cases positive for GADA only (n = 669), and LADA cases positive for both GADA and IA2A (n = 309). Approximated odds ratios were calculated using *μ* (intercept) and *β* (effect size) estimates from the linear mixed model, with the formula: $\mathrm{OR}=\frac{\beta}{e^{\mu \left(1-\mu \right)}}$ [18].

### Genetic risk scores (GRS)

We calculated two GRS using 69 T1D and T2D SNPs for T1D cases (n = 1990), T2D cases (n = 1960), LADA cases (n = 978), LADA cases positive for GADA only (n = 669), LADA cases restricted on GADA+ IA2A+ status (n = 309), and BMDCS controls (n = 1057). Weights utilized for the scores were derived from published odds ratios (ORs) from T1Dbase (t1dbase.org) or a previous publication [19], respectively. Two SNPs, rs2187668 and rs7454108, were used to infer HLA DR3/DR4/DQ8 haplotypes, and additional HLA SNPs tagging HLA A, HLA B, and DRB1 haplotypes were included [6, 20]. rs7111341 and rs11171710 did not have publicly available ORs, and rs7202877 is implicated in both T1D and T2D (Additional file 1: Table S1), so these were excluded. Each GRS was calculated using PLINK by multiplying the number of risk-increasing alleles by the natural log of the OR at each locus and summing across risk loci for each individual. Logistic regression and receiver operating characteristic (ROC) curve analyses evaluated how well these GRS distinguished LADA cases from BMDCS controls (using the PredictABEL package [21]). We repeated the GRS calculation for GADA+ and IA2+ LADA cases and for GADA+, IA2A– LADA cases. Additionally, we combined the T1D and T2D SNPs (139 SNPs) and classified LADA and controls for both LADA groups. The distributions of the T1D and T2D GRS of the five groups were compared using the Wilcoxon rank sum test accounting for multiple comparisons (using a Bonferroni correction). Control samples were obtained from the WTCCC2 study, as described above.

## Results

### T1D loci

Four T1D SNPs were significantly associated with LADA and survived multiple testing correction (*P* = 0.05/67, loci tested = 7.46 × 10^–4^; Table 1 and Additional file 1: Table S2). The strongest association was at the MHC region (OR = 1.46; *P =* 9.64 × 10^–11^). Strong association was also observed for variants at *PTPN22* (OR = 1.47; *P* = 6.38 × 10^–6^), *SH2B3* (OR = 1.28; *P* = 1.10 × 10^–5^), and *INS* (OR = 1.27; *P* = 2.39 × 10^–4^). The association signal within the MHC region was significantly different between LADA and T1D cases (*P*_difference_ = 1.26 × 10^–17^), with the T1D risk allele of rs9272346 (A) less common in LADA than in T1D, but still at a higher frequency than in controls. The signals at *INS* and *SMARCE1* also yielded significant differences between LADA and T1D (*P*_difference_ = 3.88 × 10^–4^ and 6.54 × 10^–4^, respectively). The *INS* signal was more common in LADA than in either T1D or controls, while the frequency of the *SMARCE1* signal was lower in LADA than in T1D but similar to controls.

**Table 1:** Association of established type 1 diabetes (T1D) loci with latent autoimmune diabetes in adulthood (LADA). Only T1D variants significantly associated with LADA are shown (LADA association *P* value), as well as signals significantly different between LADA and T1D (LADA vs. T1D *P* value), with a significance threshold of *P* = 7.46 × 10^–4^. The locus reported is the closest gene of interest to the signal (a full list of genes is provided in Additional file 1: Table S2). The risk and other alleles reported refer to the alleles in T1D, and the following allele frequencies refer to the frequency of the risk allele reported in T1D for LADA, T1D, and Bone Mineral Density in Childhood Study (BMDCS) control group. Odds ratios of the risk allele reported are derived from the BMDCS control data set (n = 1057), the Wellcome Trust Case Control Consortium T1D (n = 1990), and the LADA cases (n = 978)

Locus	SNP	T1D alleles risk/other	Risk allele frequency			LADA odds ratio	LADA *P* value	LADA vs. T1D *P* value
			LADA	T1D	Control			
*MHC*	rs9272346	A/G	0.686	0.818	0.579	1.455 (1.427–1.483)	9.6 × 10^–11^	1.26 × 10^–17^
*PTPN22*	rs6679677	A/C	0.143	0.17	0.093	1.469 (1.427–1.510)	6.38 × 10^–6^	2.61 × 10^–2^
*SH2B3*	rs17696736	G/A	0.515	0.503	0.44	1.277 (1.250–1.304)	1.10 × 10^–5^	0.542
*INS*	rs689	T/A	0.796	0.741	0.73	1.265 (1.234–1.296)	2.39 × 10^–4^	3.88 × 10^–4^
*SMARCE1*	rs7221109	C/T	0.621	0.687	0.632	0.954 (0.925–0.983)	0.423	6.54 × 10^–4^

To further understand the influence of antibody status on the clinical classification of LADA, the same analyses were carried out for 669 GADA+ LADA subjects (Additional file 1: Table S3). The MHC region was the only signal surviving correction for multiple comparison for cases against controls, as well as cases versus T1D (OR = 1.30; *P* = 6.84 × 10^-5^, *P*_difference_ = 1.99 × 10^–24^).

In the restricted subset of GADA+ IA2A+ LADA cases (n = 309), four loci were associated (Table 2 and Additional file 1: Table S4). The MHC (OR = 1.98; *P* = 1.20 × 10^–15^), *PTPN22* (OR = 1.86; *P* = 2.19 × 10^–6^), *SH2B3* (OR = 1.48; *P* = 5.93 × 10^–6^), and *INS* (rs689; OR = 1.44; *P* = 1.90 × 10^–4^) signals remained strongly associated and had stronger ORs in this constrained setting. However, the risk-increasing allele at the MHC locus remained significantly less than that in T1D cases. Two partially independent signals near *INS* (r^2^ = 0.278) yielded a significant difference between T1D and GADA+ IA2A+ LADA in this restricted dataset, rs689 (*P*_difference_ = 1.68 × 10^–6^) and rs7111341 (*P*_difference_ = 2.39 × 10^–4^).

**Table 2:** Association of established type 1 diabetes (T1D) loci in latent autoimmune diabetes in adulthood (LADA) subjects positive for both glutamic acid decarboxylase autoantibodies and insulinoma-associated antigen-2 autoantibodies. Only T1D variants significantly associated with LADA are shown (LADA association *P* value), as well as signals significantly different between LADA and T1D (LADA vs. T1D *P* value). Significance threshold is 7.46 × 10^–4^ after correcting for multiple comparison. The locus reported is the closest, well-known gene of interest to the signal (a full list of genes is provided in Additional file 1: Table S3). The risk and other alleles reported refer to the alleles in T1D, and the following allele frequencies refer to the frequency of the risk allele reported in T1D for LADA, T1D, and Bone Mineral Density in Childhood Study (BMDCS) control group. Odds ratios of the risk allele reported are derived from the BMDCS control data set (n = 1057), the Wellcome Trust Case Control Consortium T1D (n = 1990), and the constrained N LADA cases (n = 309). *Independent signals (*INS* signals have an r^2^ = 0.278)

Locus	SNP	T1D alleles risk/other	Risk allele frequency			LADA odds ratio	LADA *P* value	LADA vs. T1D *P* value
			LADA	T1D	Control			
*MHC*	rs9272346	A/G	0.763	0.818	0.579	1.983 (1.954–2.012)	1.20 × 10^–15^	4.01 × 10^–3^
*PTPN22*	rs6679677	A/C	0.17	0.17	0.093	1.864 (1.819–1.909)	2.19 × 10^–6^	0.603
*SH2B3*	rs17696736	G/A	0.542	0.503	0.44	1.481 (1.452–1.511)	5.93 × 10–6	0.180
INS*	rs689	T/A	0.824	0.741	0.73	1.440 (1.407–1.474)	1.90 × 10^–4^	1.68 × 10^–6^
INS*	rs7111341	C/T	0.812	0.75	0.73	1.360 (1.327–1.394)	1.82 × 10^–3^	2.39 × 10^–4^

### T2D loci

Only one T2D signal survived correction for multiple comparisons (*P* = 0.05/71, loci = 7.04 × 10^–4^) in LADA cases, the *HNF1A* locus (OR = 1.291; *P* = 3.42 × 10^–4^; Table 3 and Additional file 1: Table S5). Contrary to previous reports [12, 14, 15], the T2D risk allele (rs7903146-T) at *TCF7L2* was not enriched among LADA cases, with a frequency close to that of controls (0.295 vs. 0.298, respectively); indeed, the *TCF7L2* signal was the most significantly different signal between LADA and T2D cases (*P*_difference_ = 5.21 × 10^–6^). In the GADA+ restricted set, there were no association signals surviving correction for multiple comparisons, and the only signal showing a significant difference between LADA and T2D was the depletion of the *TCF7L2* T allele (*P*_difference_ = 5.03 × 10^–4^; Additional file 1: Table S6), where the T allele showed modest, albeit non-significant excess when compared to controls (OR = 1.088).

**Table 3:** Association of established type 2 diabetes (T2D) loci with latent autoimmune diabetes in adulthood (LADA). Only T2D variants significantly associated with LADA after correcting for multiple comparison (*P* < 7.04 × 10^–4^) are shown (LADA association *P* value), as well as variants significantly different between LADA and T2D (LADA vs. T2D *P* value). The locus reported is the closest, well-known gene of interest to the signal (a full list of genes are provided in Additional file 1: Table S4). The risk and other alleles reported refer to the alleles in T2D, and the following allele frequencies refer to the frequency of the risk allele reported in T2D, for LADA, T2D, and Bone Mineral Density in Childhood Study (BMDCS) control groups. Odds ratios of the risk allele reported are derived from the BMDCS control data set (n = 1057), the Wellcome Trust Case Control Consortium T2D (n = 1960), and the LADA cases (n = 978)

Locus	SNP	T2D alleles risk/other	Risk allele frequency			LADA odds ratio	LADA *P* value	LADA vs. T2D *P* value
			LADA	T2D	Control			
*HNF1A*	rs12427353	G/C	0.831	0.828	0.787	1.291 (1.256–1.326)	3.42 × 10^–4^	0.538
*TCF7L2* ^a^	rs7903146	T/C	0.295	0.376	0.298	1.023 (0.994–1.053)	0.702	5.21 × 10^–6^

^a^Although the control risk allele frequency is greater than the case risk allele frequency, the beta calculated from the linear mixed model is adjusted effects after controlling for population stratification, resulting in an OR slightly above 1

In the restricted set of 309 GADA+ IA2A+ LADA subjects, *HNF1A* continued to yield a significant association (OR = 1.47; *P* = 2.52 × 10^–4^; Table 4 and Additional file 1: Table S7). Again, the *TCF7L2* locus was significantly different between LADA and T2D cases (*P*_difference_ = 2.56 × 10^–7^), with the risk allele frequency even less than that in controls in this restricted case set (allele frequency of 0.251 vs. 0.298 in LADA and controls, respectively).

**Table 4:** Association between established type 2 diabetes (T2D) loci in latent autoimmune diabetes in adulthood (LADA) cases positive for glutamic acid decarboxylase autoantibodies (GADA) and insulinoma-associated antigen-2 autoantibodies (IA2A). T2D variants that were significantly associated in LADA cases positive for GAD and IA2 autoantibodies (n = 309) (LADA association *P* value) are shown, as well as signals that were significantly different between LADA and T2D cases (LADA vs. T2D *P* value). The significance threshold was set to *P* < 7.04 × 10^–4^ to correct for multiple testing. The locus reported is the closest, well-known gene of interest to the signal (a full list of genes is provided in Additional file 1: Table S5). T2D risk allele frequencies reported are derived from the Bone Mineral Density in Childhood Study control data set (n = 1057), the Wellcome Trust Case Control Consortium T2D (n = 1960), and the LADA cases positive for autoantibodies GADA and IA2A (n = 309). The odds ratios for LADA are shown both GEMMA-corrected (for relatedness and batch effects) and uncorrected

Locus	SNP	T2D alleles risk/other	Risk allele frequency			LADA odds ratio	LADA *P* value	LADA vs. T2D *P* value
			LADA	T2D	Control			
*HNF1A*	rs12427353	G/C	0.857	0.828	0.787	1.474 (1.438–1.511)	2.52 × 10^–4^	5.42 × 10^–2^
*ZBED3*	rs6878122	A/G	0.744	0.658	0.706	1.216 (1.184–1.249)	3.86 × 10^–2^	1.47 × 10^–5^
*TCF7L2*	rs7903146	T/C	0.251	0.376	0.298	0.852 (0.820–0.883)	8.14 × 10^–2^	2.56 × 10^–7^

Leveraging 2820 healthy adult British subjects from the WTCCC as alternative controls, we observed very consistent results overall (Additional file 1: Table S8 and Supplementary Results) despite the array differences for this set.

### GRS

**Fig. 1:**
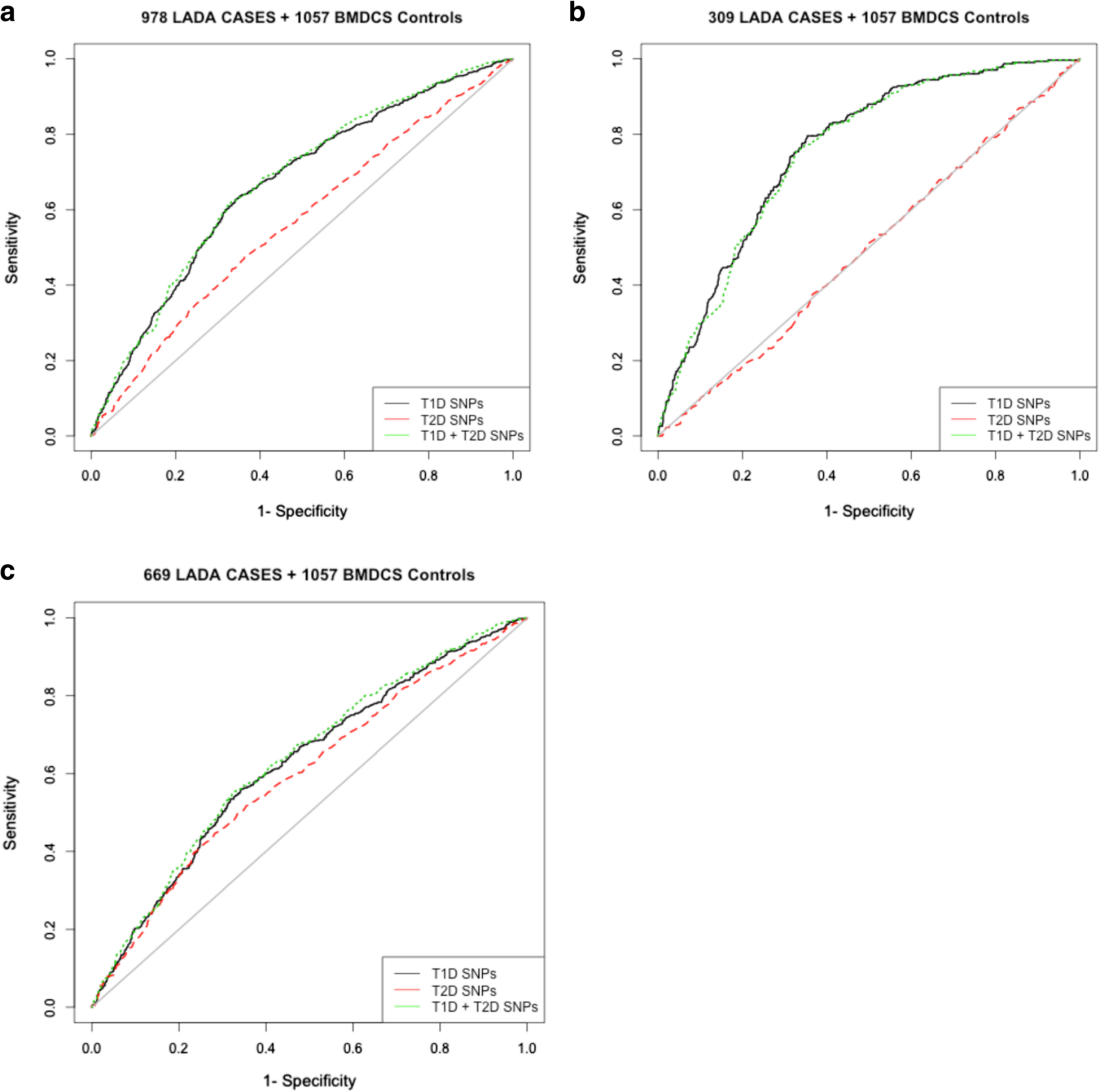
Type 1 (T1D) and type 2 diabetes (T2D) genetic risk scores (GRS) tested in latent autoimmune diabetes in adulthood (LADA) cases and controls. Weighted GRS for T1D (black) and T2D (red) were calculated by summing over all the risk alleles (T1D/T2D SNPs). The scores were tested in **a** 978 LADA cases and 1057 healthy controls; **b** 309 autoantibody-positive (glutamic acid decarboxylase autoantibodies (GADA) and insulinoma-associated antigen-2 autoantibodies (IA2A)) LADA cases and 1057 controls; **c** 669 GADA-only autoantibody positive. The ability of the GRS to discriminate between cases and controls was assessed by receiver and operator characteristic analysis. The area under the curve (AUC) was 0.667 and 0.565 for T1D and T2D, respectively, in the set with all LADA cases, 0.76 for T1D and 0.496 for T2D in the GADA, IA2A autoantibody-positive restricted set, and 0.623 for T1D, 0.597 for T2D in the GADA-only autoantibody-positive restricted set. A combination of T1D and T2D SNPs (green) had an AUC of 0.673 for all samples, 0.755 for the GADA+ IA2A+ restricted set, and 0.635 for the GADA-only restricted set

A high T1D GRS implies a high genetic risk for that disease. Figure 1 shows that the T1D GRS better predicted whether a subject is a LADA case or control than the T2D GRS. The areas under the curve (AUC) for the T1D and T2D GRS were 0.667 and 0.565, respectively (Fig. 1a). Thus, when considering adult-onset diabetes patients who do not initially require insulin, genetic risk defined for T1D could better identify autoimmune diabetes cases than genetic risk defined for T2D.

This result was more pronounced when considering controls versus 309 GADA+ IA2A+ LADA cases (Fig. 1b) (AUC for T1D GRS = 0.760, T2D GRS = 0.496). However, these results were less pronounced for the 669 GADA+ only LADA cases versus controls (AUC for T1D GRS = 0.623, T2D GRS = 0.597). The combined effect of genetic risk using both T1D and T2D SNPs marginally improved classification of LADA cases and controls (AUC = 0.673) and classification of GADA+ LADA and controls (AUC = 0.635). However, there was no improvement of classification between GADA+ IA2A+ LADA and controls (AUC = 0.755) using a combination of T1D-T2D SNPs. To highlight the important role of non-HLA loci in discrimination, we calculated T1D GRS without the HLA region and an HLA only GRS. Additionally, we tested these five models of GRS in discrimination between the LADA categories versus T1D and GADA+ only LADA cases versus GADA + IA2A+ LADA cases (Additional file 1: Figure S3). The HLA alone accounted for a strong difference between all LADA cases and T1D cases (AUC = 0.699), especially between T1D and GADA+ only LADA cases (AUC = 0.733). The non-HLA GRS had an AUC of 0.655 for distinguishing GADA + IA2A+ LADA cases from controls. The HLA-only GRS had an AUC of 0.737 for distinguishing GADA + IA2A+ LADA cases from controls, but combining these loci, the AUC was 0.76.

**Fig. 2:**
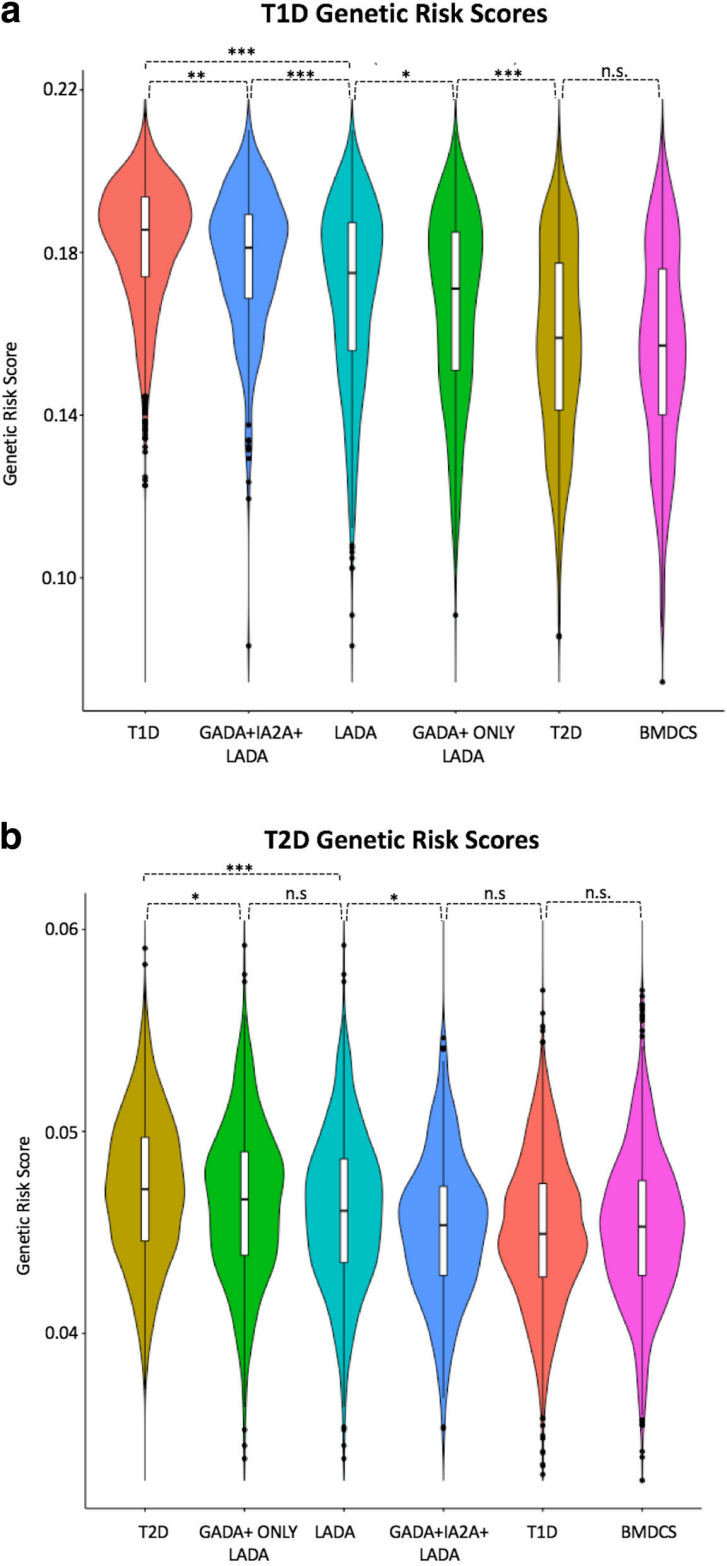
Genetic risk score (GRS) distributions between type 1 diabetes (T1D), type 2 diabetes (T2D), latent autoimmune diabetes in adulthood (LADA), and LADA-restricted cases and controls. The GRS distributions were compared across individuals diagnosed with T1D (n = 1990), T2D (n = 1960), LADA (n = 978), LADA restricted (n = 309), LADA GADA-only (n = 669), and Bone Mineral Density in Childhood Study controls (n = 1057). **a** Violin plots of the distributions of the GRS calculated using the T1D SNPs for the five groups. A multiple comparison test (Wilcoxon rank sum test) was performed to calculate the significance of pair-wise differences. **b** Violin plots of the distributions of the GRS calculated using the T2D SNPs for the five groups. A multiple comparison test (Wilcoxon rank sum test) was performed to calculate the significance of pair-wise differences. We include some of the significant *P* values to highlight key differences. (^***^ *P* < 10^–5^, ^**^ *P* < 0.0001, ^*^ *P* < 0.05)

Comparison of the T1D SNP-GRS distributions among the six groups (T1D, T2D, LADA, GADA+ IA2A+ LADA, GADA-only LADA, and controls; Fig. 2a) revealed significant differences between all pairs (*P* < 10^–5^), except T2D versus controls. This observation was as expected as T2D cases should not harbor a high load of T1D risk alleles. Furthermore, there were only nominally significant differences between LADA and GADA-only LADA cases. Of particular note, there was a significant difference in the T1D GRS distribution between T1D and GADA+ IA2A+ LADA, highlighting genetic differences between LADA restricted on IA2A+ status and T1D (*P* = 0.0001).

Comparison of the distributions of the T2D SNP-GRS (Fig. 2b) revealed significant differences between LADA and T2D cases (*P* = 3.50 × 10^–11^) and between the GADA+ IA2A+ LADA and T2D cases (*P* = 3.50 × 10^–16^). These results suggest T2D risk alleles are not enriched in LADA, concordant with the results of our single-SNP analyses. However, the T2D SNP-GRS distribution was also significantly different between LADA and T1D cases (*P* = 6.10 × 10^–11^) and controls (*P* = 8.00 × 10^–6^). The T2D risk allele load, although not as high as for T2D, is still higher than that seen in T1D or among the healthy population. We observed a nominally significant difference for T2D risk allele load between GADA+ only LADA and T2D cases (*P* = 5.60 × 10^–3^) and no statistically significant difference between GADA+ only LADA and overall LADA cases.

## Discussion

Defining LADA as a distinct form of T1D has two broad benefits. First, it highlights the potential to understand what determines both the degree and rate of disease progression. Second, it helps define differences between adult-onset autoimmune diabetes, including LADA, and T2D in terms of co-morbidities and putative therapy [22]. Leveraging children whose future diabetes risk is unknown represents the most conservative setting in which to conduct this study given they serve as excellent population-based controls in which to contrast the cases; however, the conservative nature of the approach may result in some false negative results.

To shed light on the genetic etiology of LADA, we tested the impact of established T1D and T2D risk loci in the largest set of LADA cases collected to date. Our study differs from a previous association study with GWAS-implicated loci in adult-onset autoimmune diabetes by Howson et al. [23]; first, our LADA cases are distinguished by the fact that they were not treated with insulin upon diagnosis. Furthermore, our study looked at a larger set of T1D and T2D loci, as well as comparing their roles in LADA against T1D and T2D, including taking population substructure into account. As with Howson et al. [23], we observed significant association of the T1D loci *PTPN22*, *INS*, *HLA*, and *SH2B3*. However, we did not observe significant association with the *CLEC16A*, *IL2RA*, *CTLA4*, and *STAT* loci*.* Despite published data observing the association of T2D locus *TCF7L2* with a subset of T1D patients [24, 25], our study did not observe an association of this locus with LADA; one possibility could be that we used population-based controls, while previous studies may have used a different control strategy where the difference in the risk allele was more evident due to its under-representation in relatively disease-free controls. Our study goes further by leveraging GRS to offer a further line of evidence for the classification of diabetes subtypes, complementing standards for clinical decision-making and additional standardized (antibody) testing, each with their strengths and weaknesses.

LADA shows the MHC risk found in adult-onset T1D [23] with a reduced genetic susceptibility at this locus compared with childhood-onset T1D. Less clear is whether T2D loci play a role in adult-onset autoimmune diabetes. Our results show that genetic signals implicated in T1D or T2D both play a role in LADA, with four T1D loci and one T2D locus significantly associated with this form of diabetes. LADA is genetically more similar to T1D, especially when cases are constrained on both GADA+ and IA2A+, although LADA shares part of its genetic etiology with T2D. When constrained on GADA+ only, LADA cases became less distinct from T2D, highlighting the importance of IA2A in discriminating LADA within the T1D-T2D spectrum. By implication, a GRS derived from T1D can discriminate, to a degree, non-insulin requiring adult-onset diabetes patients with either autoimmune diabetes or T2D.

Regarding the loci implicated in T1D, our results are consistent with previous studies showing a major role for the MHC, *PTPN22*, and *INS* loci in LADA [10, 12, 13]. Interestingly, the risk allele frequency at *INS* (rs689) was even more strongly associated with LADA than with T1D. Therefore, our data strongly points to common insulin-related pathways underpinning autoimmune diabetes irrespective of the age at onset of the disease. Given the evidence that age at diagnosis is genetically determined [26], these loci may play a key role in determining the age at disease onset and the rate of disease progression.

While our results suggest LADA is genetically closer to T1D than to T2D, we observed an association at one T2D locus, *HNF1A*, known to be associated with T2D and ‘maturity-onset diabetes of the young’; strikingly, the *HNF1A* signal remained significantly associated with LADA even in the cohort enriched for both T1D autoantibodies. Nevertheless, the nature of the role of *HNF1A* in LADA is unclear, although any gene compromising insulin secretory function could predispose to diabetes. This is the first report describing an association between this T2D-associated risk allele and LADA, although this locus has been previously implicated in T1D [16]. Additionally, the strongest T2D-associated locus, *TCF7L2*, has been associated with LADA in a Finnish cohort [14–16], but in our study, the risk allele frequency in LADA was very close to that of controls and lower than controls in GADA+ IA2A+ LADA. Our findings were further supported by leveraging healthy adult British controls from the WTCCC, which provided overall consistent results, including for the *HNF1A* signal. However, given the borderline association of T2D loci identified and the modest power in this single study, these signals must be subjected to replication efforts by independent investigators in order to fully validate these observations.

We found that, from GRS calculated from T1D- and T2D-implicated SNPs, which distinguished LADA cases from controls, the T1D GRS performed better than the T2D GRS; this difference was particularly striking in GADA+ IA2A+ LADA cases. Comparison of GRS between the six defined groups placed LADA in between T1D and T2D but closer to T1D. GADA+ IA2A+ LADA was very similar to T1D, primarily because such constraint filters out ‘T2D-like’ cases and enriches for ‘T1D-like’ cases. The potential for clinical, immunological, or genetic filters to define forms of diabetes is emphasized by the marked overlap in GRS scores, even between T1D and controls.

This study does have limitations. First, GADA-only LADA cases had a T2D-SNP GRS distribution more similar to T2D than controls. The specific association between the T2D risk score and GADA-only LADA cases could be in part due to the fact that a fraction of these cases might be false antibody-positive T2D, though those with double antibody positivity are likely to have a very low false positive rate. Thus, larger studies may resolve whether T2D risk alleles play a role in LADA. Indeed, this study was underpowered to identify specific associations other than for *HNF1A*. Second, two different genotyping arrays were utilized; thus, to correct for potential batch effects due to genotype array differences, population substructure, and relatedness among samples, we used a linear mixed model, resulting in highly conservative effect estimates. Consequently, it is possible that we have missed some true positive associations since we robustly controlled for false positive results.

The current nomenclature to classify diabetes, designating it as ‘T1D’ or ‘T2D’, was adopted to foster research and appropriate therapy for different phenotypic presentations. The combination of GRS, age at diagnosis, clinical phenotype, autoantibody assays, and C-peptide estimates as a proxy for insulin secretion affords a more sophisticated approach with the potential to dissect the heterogeneity of diabetes [6]. This study highlights the uncertainty of the current classification of diabetes [27]. These results suggest that clinical phenotype alone is insufficient to define the major types of diabetes. To better treat the various diabetes subtypes, we need to integrate the use of clinical phenotype, metabolic status, immune changes, and underlying genetic risk.

## Conclusion

LADA is genetically closer to T1D than T2D, although the genetic load of T1D risk alleles is less than childhood-onset T1D, particularly at the MHC, potentially accounting for the later disease onset. Our results show that the genetic spectrum of T1D extends into adult-onset diabetes, where it can clinically masquerade as T2D. Furthermore, T2D genetic risk plays a small role in LADA, with a degree of evidence for the *HNF1A* locus, highlighting the potential for GRS to contribute towards defining diabetes subtypes.

## Additional file

Additional file 1: Supplementary material. **Table S1.** Established SNPs for GRS. **Table S2.** Association of established T1D loci with LADA cases (*P*-value LADA vs BMDCS) and comparison to T1D cases (LADA vs. T1D *P*-value). **Table S3.** Association of established T1D loci with GADA+ only LADA cases (*P*-value LADA vs BMDCS) and comparison to T1D cases (LADA vs. T1D *P*-value). **Table S4.** Association of established T1D loci with GADA+,IA2A+ LADA cases and comparison to T1D cases using controls. **Table S5.** Association of established T2D loci with LADA cases (*P*-value LADA vs BMDCS) and comparison to T2D cases (LADA vs. T2D *P*-value). **Table S6.** Association of established T2D loci with GADA+ only LADA cases (*P*-value LADA vs BMDCS) and comparison to T2D cases (LADA vs. T2D *P*-value). **Table S7.** Association of established T2D loci with GADA+,IA2A+ LADA cases in comparison to association in T2D cases using controls. Supplementary Results. Follow-up analysis using WTCCC as an alternative control source. **Table S8.** Table of results for follow-up analysis using WTCCC as an alternative control source. **Figure S1.** Principle component analysis of LADA cases & controls. **Figure S2.** Quantile-quantile plot of pruned markers used for PCs for LADA cases and controls. **Figure S3.** Extended set of ROC plots including Non-HLA and HLA only GRS also tested in LADA vs T1D and GADA+ only LADA vs GADA+,IA2A+ LADA. Weighted genetic risk scores (GRS) for T1D GRS (black), T1D without HLA GRS (blue) , HLA only GRS (cyan), T2D GRS (red) and both T1D and T2D GRS (green) were calculated and tested in A) LADA cases and controls B) GADA+ only and controls C) GADA+,IA2A+ LADA and controls D) LADA and WTCCC T1D cases E) GADA+ only LADA and WTCCC T1D F) GADA+,IA2A+ LADA and WTCCC T1D. Supplemental Methods. More details on recruitment of LADA cases and genotyping methods. (DOCX 1592 kb)
