# Supplementary material for: Relative contribution of type 1 and type 2 diabetes loci to the genetic etiology of adult-onset, non-insulin-requiring autoimmune diabetes
Source: BMC Med. 2017 Apr 25;15:88. doi: 10.1186/s12916-017-0846-0 (PMC5404312; doi:10.1186/s12916-017-0846-0)
Supplement: Supplementary file 1 — Supplementary material. Table S1. Established SNPs for GRS. Table S2. Association of established T1D loci with LADA cases (P-value LADA vs BMDCS) and comparison to T1D cases (LADA vs. T1D P-value). Table S3. Association of established T1D loci with GADA+ only LADA cases (P-value LADA vs BMDCS) and comparison to T1D cases (LADA vs. T1D P-value). Table S4. Association of established T1D loci with GADA+,IA2A+ LADA cases and comparison to T1D cases using controls. Table S5. Association of established T2D loci with LADA cases (P-value LADA vs BMDCS) and comparison to T2D cases (LADA vs. T2D P-value). Table S6. Association of established T2D loci with GADA+ only LADA cases (P-value LADA vs BMDCS) and comparison to T2D cases (LADA vs. T2D P-value). Table S7. Association of established T2D loci with GADA+,IA2A+ LADA cases in comparison to association in T2D cases using controls. Supplementary Results. Follow-up analysis using WTCCC as an alternative control source. Table S8. Table of results for follow-up analysis using WTCCC as an alternative control source. Figure S1. Principle component analysis of LADA cases & controls. Figure S2. Quantile-quantile plot of pruned markers used for PCs for LADA cases and controls. Figure S3. Extended set of ROC plots including Non-HLA and HLA only GRS also tested in LADA vs T1D and GADA+ only LADA vs GADA+,IA2A+ LADA. Weighted genetic risk scores (GRS) for T1D GRS (black), T1D without HLA GRS (blue) , HLA only GRS (cyan), T2D GRS (red) and both T1D and T2D GRS (green) were calculated and tested in A) LADA cases and controls B) GADA+ only and controls C) GADA+,IA2A+ LADA and controls D) LADA and WTCCC T1D cases E) GADA+ only LADA and WTCCC T1D F) GADA+,IA2A+ LADA and WTCCC T1D. Supplemental Methods. More details on recruitment of LADA cases and genotyping methods. (DOCX 1592 kb) [file 12916_2017_846_MOESM1_ESM.docx]

**Additional file 1**

**Table of Contents**

| **Table S1** | Established SNPs for GRS Calculation | Pages 2-3 |
| --- | --- | --- |
| **Table S2** | LADA (n= 978) vs T1D (BMDCS controls) | Pages 4-5 |
| **Table S3** | LADA cases positive for GADA only (n= 669) vs T1D (BMDCS controls) | Pages 6-7 |
| **Table S4** | LADA cases positive for GADA and IA2A (n= 309) vs T1D (BMDCS controls) | Pages 8-9 |
| **Table S5** | LADA (n= 978) vs T2D (BMDCS controls) | Page 10 |
| **Table S6** | LADA cases positive for GADA only (n= 669) vs T2D (BMDCS controls) | Page 11 |
| **Table S7** | LADA cases positive for GADA and IA2A (n= 309) vs T2D (BMDCS controls) | Page 12 |
| **Supplementary Results** | Descriptive results for follow-up analysis using WTCCC as an alternative control source | Page 13 |
| **Table S8** | Table of results for follow-up analysis using WTCCC as an alternative control source | Pages 14-15 |
| **Figure S1** | Principle component analysis of LADA cases & BMDCS controls | Pages 16-17 |
| **Figure S2** | QQ-plot and genomic inflation factor | Page 18 |
| **Figure S3** | ROC Curve with Non-HLA and HLA only GRS models | Page 19 |
| **Supplementary Methods** | More details on recruitment of LADA cases and genotyping methods | Pages 20-22 |

**Table S1: Established SNPs for GRS Calculation**

| T1D SNPS | Gene | Odds Ratio | Weight | Effect Allele |
| --- | --- | --- | --- | --- |
| rs2187668, rs7454108 | DR3/DR4-DQ8 | 48.18 | 3.87 |  |
|  | DR3/DR3 | 21.12 | 3.05 |  |
|  | DR4-DQ8/DR4-DQ8 | 21.98 | 3.09 |  |
|  | DR4-DQ8/X | 7.03 | 1.95 |  |
|  | DR3/X | 4.53 | 1.51 |  |
| rs1264813 | HLA_A_24 | 1.54 | 0.43 | T |
| rs2395029 | HLA_B_5701 | 2.51 | 0.92 | T |
| rs3129889 | HLA_DRB1_15 | 14.88 | 2.7 | A |
| rs10272724 | *IKZF1, FIGNL1, DDC, GRB10* | 1.15 | 0.14 | T |
| rs10492166 | *CD69* | 1.15 | 0.14 | G |
| rs10509540 | *RNLS* | 1.14 | 0.13 | T |
| rs10517086 | *SLC34A2, SEL1L3, SMIM20, RBPJ, CCKAR, TBC1D19* | 1.09 | 0.09 | A |
| rs10795791 | *IL2RA, RBM17* | 1.16 | 0.15 | G |
| rs11170466 | *ITGB7* | 1.19 | 0.17 | T |
| rs11203203 | *UBASH3A* | 1.14 | 0.13 | A |
| rs113010081 | *CCR5* | 1.19 | 0.17 | T |
| rs11571316 | *CTLA4, ICOS* | 1.22 | 0.2 | G |
| rs11755527 | *BACH2* | 1.13 | 0.12 | G |
| rs11954020 | *IL7R* | 1.11 | 0.1 | G |
| rs12148472 | *CHRNB4, ADAMTS7, MORF4L1, CTSH, RASGRF1* | 1.20 | 0.18 | T |
| rs12453507 | *FBXL20, MED1, CDK12, NEUROD2, PPP1R1B, STARD3, TCAP, PNMT, PGAP3, ERBB2, MIEN1, GRB7, IKZF3, ZPBP2, GSDMB, ORMDL3, LRRC3C, GSDMA, PSMD3, CSF3, MED24, THRA* | 1.11 | 0.1 | G |
| rs12708716 | *CLEC16A,CIITA, DEXI, RMI2, SOCS1, TNP2, PRM3, PRM2, PRM1, CTD-3088G3.8* | 1.23 | 0.21 | A |
| rs12908309 | *RASGRP1* | 1.19 | 0.17 | G |
| rs1456988 | *C14orf64* | 1.12 | 0.11 | G |
| rs1538171 | *CENPW* | 1.12 | 0.11 | G |
| rs1615504 | *DOK6, CD226* | 1.13 | 0.12 | T |
| rs1738074 | *RSPH3, TAGAP* | 1.09 | 0.09 | C |
| rs17696736 | *SH2B3,NAA25, CUX2, FAM109A, ATXN2, BRAP, ACAD10, RP11-162P23.2 (ENSG00000257767), ALDH2, MAPKAPK5, TMEM116, ERP29, TRAFD1, HECTD4, RPL6, PTPN11, RPH3A* | 1.34 | 0.29 | G |
| rs193778 | *CLEC16A,CIITA, DEXI, RMI2, SOCS1, TNP2, PRM3, PRM2, PRM1, CTD-3088G3.8* | 1.14 | 0.13 | G |
| rs1990760 | *GCG, FAP, IFIH1, GCA, KCNH7* | 1.15 | 0.14 | T |
| rs2281808 | *SIRPD, RP4-576H24.4 (ENSG00000260861), SIRPB1, SIRPG* | 1.11 | 0.1 | C |
| rs2304256 | *ICAM1, ICAM4, ICAM5, ZGLP1, FDX1L, FDX1L, CTD-2369P2.12, RAVER1, ICAM3, TYK2, CDC37, PDE4A, KEAP1, S1PR5* | 1.16 | 0.15 | C |
| rs3024493 | *MAPKAPK2, IL10, IL19, IL20* | 1.22 | 0.2 | C |
| rs3825932 | *CHRNB4, ADAMTS7, MORF4L1, CTSH, RASGRF1* | 1.16 | 0.15 | T |
| rs402072 | *DACT3, PRKD2, STRN4, FKRP, SLC1A5* | 1.15 | 0.14 | T |
| rs4763879 | *CD69, KLRB1, CLEC2D, CLECL1* | 1.09 | 0.09 | A |
| rs478222 | *DNMT3A,CENPO, ADCY3, DNAJC27, EFR3B, POMC* | 1.15 | 0.14 | A |
| rs4849135 | *ACOXL,LINC00116, LIMS3, LIMS3L, RGPD6, BUB1, ACOXL* | 1.12 | 0.11 | G |
| rs4948088 | *COBL* | 1.30 | 0.26 | C |
| rs5753037 | *NF2, CABP7, ZMAT5, UQCR10, ASCC2, MTMR3, HORMAD2, LIF, OSM* | 1.11 | 0.1 | T |
| rs597325 | *BACH2* | 1.19 | 0.17 | G |
| rs601338 | *FUT2,SULT2B1, FAM83E, SPACA4, RPL18, SPHK2, DBP, CA11, NTN5, MAMSTR, RASIP1, IZUMO1, FUT1, FGF21* | 1.34 | 0.29 | A |
| rs602662 | *FUT2,SULT2B1, FAM83E, SPACA4, RPL18, SPHK2, DBP, CA11, NTN5, MAMSTR, RASIP1, IZUMO1, FUT1, FGF21* | 1.12 | 0.11 | A |
| rs61839660 | *IL2RA, RBM17, PFKFB3* | 1.62 | 0.48 | C |
| rs6476839 | *GLIS3* | 1.12 | 0.11 | T |
| rs6679677 | *PTPN22, PHTF1* | 1.82 | 0.6 | A |
| rs6691977 | *KIF14, DDX59, CAMSAP2, GPR25, C1orf106* | 1.13 | 0.12 | C |
| rs689 | *INS,IGF2, INS-IGF2,TH* | 2.39 | 0.87 | T |
| rs6920220 | *TNFAIP3* | 1.12 | 0.11 | A |
| rs705705 | *IKZF4,PMEL, CDK2, RAB5B, SUOX, RPS26, ERBB3, RP11-603J24.9, PA2G4, RPL41, ZC3H10, ESYT1, MYL6B, MYL6, SMARCC2, RNF41, NABP2, SLC39A5, ANKRD52, COQ10A, CS, RP11-977G19.10, CNPY2, PAN2, IL23A, STAT2, APOF* | 1.25 | 0.22 | C |
| rs7090530 | *IL2RA, RBM17, PFKFB3* | 1.22 | 0.2 | A |
| rs7221109 | *CCR7, SMARCE1, KRT222, KRT222, KRT24* | 1.05 | 0.05 | C |
| rs72727394 | *RASGRP1, C15orf53* | 1.15 | 0.14 | T |
| rs72928038 | *BACH2* | 1.20 | 0.18 | A |
| rs7804356 | *SKAP2, C7orf71, HOXA1, HOXA2, HOXA3, HOXA4, HOXA5, HOXA6, HOXA7, HOXA9* | 1.14 | 0.13 | T |
| rs7928968 | *INS,IGF2, INS-IGF2,TH* | 1.25 | 0.22 | T |
| rs911263 | *RAD51B, ZFP36L1* | 1.12 | 0.11 | T |
| rs924043 | *WDR27, C6orf120, PHF10, TCTE3, ERMARD, DLL1, FAM120B, PSMB1, TBP, PDCD2* | 1.19 | 0.17 | C |
| rs9272346 | *MHC* | 5.58 | 1.72 | A |
| rs9585056 | *UBAC2, GPR18, GPR183, TM9SF2* | 1.12 | 0.11 | C |
| rs9924471 | *SBK1, NPIPB6, EIF3CL, NPIPB7, CLN3, CLN3, APOBR, IL27, NUPR1, CCDC101, SULT1A2, SULT1A1, NPIPB8, EIF3C, NPIPB9, ATXN2L, TUFM, SH2B1, ATP2A1, RABEP2, CD19, NFATC2IP, SPNS1, LAT* | 1.25 | 0.22 | A |
| rs10877012 | *CYP27B1* | 1.22 | 0.2 | G |
| rs11258747 | *PRKCQ* | 1.45 | 0.37 | G |
| rs1465788 | *RAD51B, ZFP36L1* | 1.16 | 0.15 | C |
| rs229541 | *IL2RB, C1QTNF6, SSTR3, RAC2* | 1.12 | 0.11 | A |
| rs2542151 | *PTPN2* | 1.30 | 0.26 | G |
| rs2611215 | *TMEM192, KLHL2, MSMO1, CPE, TLL1* | 1.19 | 0.17 | A |
| rs6827756 | *KIAA1109, ADAD1, IL2, IL21* | 1.13 | 0.12 | T |
| rs694739 | *BAD,MACROD1, FLRT1, STIP1, FERMT3, TRPT1, NUDT22, DNAJC4, VEGFB, FKBP2, PPP1R14B, PLCB3, GPR137, KCNK4, TEX40, ESRRA, TRMT112, PRDX5, CCDC88B, RPS6KA4* | 1.05 | 0.05 | A |
| rs722988 | *ITGB1, NRP1* | 1.11 | 0.1 | C |
| rs941576 | *DLK1* | 1.14 | 0.13 | A |
| rs9653442 | *AFF3* | 1.11 | 0.1 | C |
| **Type 2 Diabetes SNP** | **Gene** | **Odds Ratio** | **Weight** | **Effect Allele** |
| rs7903146 | *TCF7L2* | 1.40 | 0.34 | T |
| rs17791513 | *TLE4* | 1.21 | 0.19 | A |
| rs7756992 | *CDKAL1* | 1.20 | 0.18 | G |
| rs10811661 | *CDKN2A/B* | 1.19 | 0.17 | T |
| rs3802177 | *SLC30A8* | 1.16 | 0.15 | G |
| rs2261181 | *HMGA2* | 1.16 | 0.15 | T |
| rs1111875 | *HHEX/IDE* | 1.15 | 0.14 | C |
| rs10203174 | *THADA* | 1.15 | 0.14 | C |
| rs9936385 | *FTO* | 1.13 | 0.12 | C |
| rs6878122 | *ZBED3* | 1.13 | 0.12 | G |
| rs4430796 | *HNF1B* | 1.13 | 0.12 | G |
| rs4402960 | *IGF2BP* | 1.13 | 0.12 | T |
| rs17168486 | *DGKB* | 1.13 | 0.12 | T |
| rs1552224 | *ARAP1 (CENTD2)* | 1.13 | 0.12 | A |
| rs10401969 | *CILP2* | 1.13 | 0.12 | C |
| rs849135 | *JAZF1* | 1.12 | 0.11 | G |
| rs12427353 | *HNF1A* | 1.12 | 0.11 | G |
| rs7593730 | *RBMS1* | 1.11 | 0.1 | C |
| rs10830963 | *MTNR1B* | 1.11 | 0.1 | G |
| rs7612463 | *UBE2E2* | 1.11 | 0.1 | C |
| rs516946 | *ANK1* | 1.11 | 0.1 | C |
| rs17106184 | *FAF1* | 1.11 | 0.1 | G |
| rs1359790 | *SPRY2* | 1.11 | 0.1 | G |
| rs13233731 | *KLF14* | 1.11 | 0.1 | G |
| rs11063069 | *CCND2* | 1.11 | 0.1 | G |
| rs10923931 | *NOTCH2* | 1.11 | 0.1 | T |
| rs7955901 | *TSPAN8* | 1.09 | 0.09 | C |
| rs4458523 | *WFS1* | 1.09 | 0.09 | G |
| rs2943640 | *IRS1* | 1.09 | 0.09 | C |
| rs243088 | *BCL11A* | 1.09 | 0.09 | T |
| rs163184 | *KCNQ1* | 1.09 | 0.09 | G |
| rs12899811 | *PRC1* | 1.09 | 0.09 | G |
| rs12571751 | *ZMIZ1* | 1.09 | 0.09 | A |
| rs11717195 | *ADCY5* | 1.09 | 0.09 | T |
| rs11634397 | *ZFAND6* | 1.09 | 0.09 | G |
| rs10842994 | *KLHDC5* | 1.09 | 0.09 | C |
| rs7845219 | *TP53INP1* | 1.08 | 0.08 | T |
| rs7178572 | *HMG20A* | 1.08 | 0.08 | G |
| rs702634 | *ARL15* | 1.08 | 0.08 | A |
| rs6813195 | *TMEM154* | 1.08 | 0.08 | C |
| rs6808574 | *LPP* | 1.08 | 0.08 | C |
| rs5215 | *KCNJ11* | 1.08 | 0.08 | C |
| rs2075423 | *PROX1* | 1.08 | 0.08 | G |
| rs12970134 | *MC4R* | 1.08 | 0.08 | A |
| rs9505118 | *SSR1/RREB1* | 1.07 | 0.07 | A |
| rs6795735 | *ADAMTS9* | 1.07 | 0.07 | C |
| rs4812829 | *HNF4A* | 1.07 | 0.07 | A |
| rs4275659 | *MPHOSPH9* | 1.07 | 0.07 | C |
| rs2796441 | *TLE1* | 1.07 | 0.07 | G |
| rs2334499 | *DUSP8* | 1.07 | 0.07 | T |
| rs8108269 | *GIPR* | 1.06 | 0.06 | G |
| rs7163757 | *C2CD4A* | 1.06 | 0.06 | C |
| rs11257655 | *CDC123* | 1.06 | 0.06 | T |
| rs7041847 | *GLIS3* | 1.05 | 0.05 | A |
| rs459193 | *ANKRD55* | 1.05 | 0.05 | G |
| rs10278336 | *GCK* | 1.05 | 0.05 | A |
| rs780094 | *GCKR* | 1.04 | 0.04 | C |
| rs3923113 | *GRB14* | 1.04 | 0.04 | A |
| rs2028299 | *AP3S2* | 1.04 | 0.04 | C |
| rs831571 | *PSMD6* | 1.03 | 0.03 | C |
| rs16861329 | *ST64GAL1* | 1.03 | 0.03 | C |
| rs7403531 | *RASGRP1* | 1.02 | 0.02 | T |
| rs3786897 | *PEPD* | 1.02 | 0.02 | A |
| rs1802295 | *VPS26A* | 1.02 | 0.02 | T |
| rs9470794 | *ZFAND3* | 1.01 | 0.01 | T |
| rs6723108 | *TMEM163* | 1.01 | 0.01 | T |
| rs6467136 | *GCC1* | 1.01 | 0.01 | A |

**Table S2: LADA (n= 978) vs T1D (BMDCS controls**

| **RSID** | **Locus** | **Chr** | **T1D Alleles Risk/Other** | **Risk Allele Frequency** | | | **LADA Odds Ratio** | **LADA *P*-value** | **LADA vs T1D *P*-value** |
| --- | --- | --- | --- | --- | --- | --- | --- | --- | --- |
|  |  |  |  | **LADA** | **T1D** | **Control** |  |  |  |
| **rs9272346** | MHC | 6 | A/G | 0.686 | 0.818 | 0.579 | 1.455 [1.427-1.483] | 9.64E-11 | 1.26E-17 |
| **rs6679677** | *PTPN22, PHTF1* | 1 | A/C | 0.143 | 0.17 | 0.093 | 1.469 [1.427-1.510] | 6.38E-06 | 2.61E-02 |
| **rs17696736** | *SH2B3, NAA25, CUX2, FAM109A,, ATXN2, BRAP, ACAD10, RP11-162P23.2 (ENSG00000257767), ALDH2, MAPKAPK5, TMEM116, ERP29, TRAFD1, HECTD4, RPL6, PTPN11, RPH3A* | 12 | G/A | 0.515 | 0.503 | 0.44 | 1.277 [1.250-1.304] | 1.10E-05 | 5.42E-01 |
| **rs689** | *INS,IGF2, INS-IGF2,TH* | 11 | T/A | 0.796 | 0.741 | 0.73 | 1.265 [1.234-1.296] | 2.39E-04 | 3.88E-04 |
| **rs597325** | *BACH2* | 6 | G/A | 0.646 | 0.647 | 0.583 | 1.192 [1.164-1.220] | 2.11E-03 | 5.06E-01 |
| **rs7804356** | *SKAP2, C7orf71, HOXA1, HOXA2, HOXA3, HOXA4, HOXA5, HOXA6, HOXA7, HOXA9* | 7 | T/C | 0.791 | 0.768 | 0.751 | 1.217 [1.185-1.249] | 2.77E-03 | 5.87E-02 |
| **rs11954020** | *IL7R* | 5 | G/C | 0.438 | 0.415 | 0.387 | 1.174 [1.146-1.202] | 5.44E-03 | 2.03E-01 |
| **rs4505848** | *KIAA1109, ADAD1, IL2, IL21* | 4 | G/A | 0.36 | 0.363 | 0.321 | 1.160 [1.131-1.189] | 1.23E-02 | 7.92E-01 |
| **rs9585056** | *UBAC2, GPR18, GPR183, TM9SF2* | 13 | C/T | 0.246 | 0.266 | 0.22 | 1.174 [1.142-1.206] | 1.54E-02 | 3.44E-02 |
| **rs11755527** | *BACH2* | 6 | G/C | 0.481 | 0.5 | 0.43 | 1.139 [1.112-1.166] | 1.75E-02 | 2.36E-01 |
| **rs229541** | *IL2RB, C1QTNF6, SSTR3, RAC2* | 22 | A/G | 0.436 | 0.468 | 0.412 | 1.136 [1.108-1.164] | 2.81E-02 | 6.70E-02 |
| **rs1538171** | *CENPW* | 6 | G/C | 0.514 | 0.475 | 0.472 | 1.127 [1.099-1.155] | 3.64E-02 | 1.38E-01 |
| **rs402072** | *DACT3, PRKD2, STRN4, FKRP, SLC1A5, AC008622.1 (ENSG00000280050)* | 19 | T/C | 0.861 | 0.862 | 0.841 | 1.173 [1.134-1.212] | 4.41E-02 | 6.13E-01 |
| **rs12908309** | *RASGRP1* | 15 | G/A | 0.779 | 0.78 | 0.76 | 1.142 [1.107-1.176] | 5.96E-02 | 3.07E-01 |
| **rs9653442** | *AFF3* | 2 | C/T | 0.485 | 0.502 | 0.454 | 1.109 [1.082-1.137] | 6.53E-02 | 2.71E-01 |
| **rs7928968** | *INS,IGF2, INS-IGF2,TH* | 11 | T/A | 0.259 | 0.267 | 0.232 | 1.144 [1.108-1.180] | 6.72E-02 | 4.27E-01 |
| **rs72928038** | *BACH2* | 6 | A/G | 0.193 | 0.21 | 0.168 | 1.162 [1.122-1.202] | 6.74E-02 | 9.66E-03 |
| **rs601338** | *FUT2,SULT2B1, FAM83E, SPACA4, RPL18, SPHK2, DBP, CA11, NTN5, MAMSTR, RASIP1, IZUMO1, FUT1, FGF21* | 19 | A/G | 0.451 | 0.456 | 0.507 | 0.905 [0.878-0.932] | 7.33E-02 | 8.10E-01 |
| **rs6476839** | *GLIS3* | 9 | T/A | 0.448 | 0.435 | 0.413 | 1.096 [1.067-1.124] | 1.14E-01 | 5.32E-01 |
| **rs911263** | *RAD51B, ZFP36L1* | 14 | T/C | 0.706 | 0.74 | 0.691 | 1.091 [1.062-1.121] | 1.49E-01 | 2.38E-01 |
| **rs602662** | *FUT2,SULT2B1, FAM83E, SPACA4, RPL18, SPHK2, DBP, CA11, NTN5, MAMSTR, RASIP1, IZUMO1, FUT1, FGF21* | 19 | G/A | 0.523 | 0.518 | 0.513 | 1.084 [1.057-1.111] | 1.49E-01 | 5.72E-01 |
| **rs2542151** | *PTPN2* | 18 | G/T | 0.148 | 0.202 | 0.166 | 0.897 [0.860-0.934] | 1.53E-01 | 2.58E-03 |
| **rs4763879** | *CD69, KLRB1, CLEC2D, CLECL1* | 12 | A/G | 0.397 | 0.392 | 0.376 | 1.085 [1.056-1.113] | 1.58E-01 | 8.13E-01 |
| **rs694739** | *BAD,MACROD1, FLRT1, STIP1, FERMT3, TRPT1, NUDT22, DNAJC4, VEGFB, FKBP2, PPP1R14B, PLCB3, GPR137, KCNK4, TEX40, ESRRA, TRMT112, PRDX5, CCDC88B, RPS6KA4* | 11 | A/G | 0.64 | 0.639 | 0.608 | 1.085 [1.056-1.113] | 1.64E-01 | 7.21E-01 |
| **rs941576** | *DLK1* | 14 | A/G | 0.574 | 0.602 | 0.56 | 1.084 [1.055-1.112] | 1.64E-01 | 3.81E-01 |
| **rs10509540** | *RNLS* | 10 | T/C | 0.741 | 0.75 | 0.716 | 1.086 [1.056-1.116] | 1.76E-01 | 7.69E-01 |
| **rs4849135** | *ACOXL,LINC00116 (ENSG00000175701), LIMS3 (ENSG00000257207), LIMS3L, RGPD6, BUB1, ACOXL* | 2 | G/T | 0.73 | 0.727 | 0.717 | 1.076 [1.045-1.107] | 2.42E-01 | 9.59E-01 |
| **rs10877012** | *CYP27B1* | 12 | T/G | 0.336 | 0.313 | 0.311 | 1.071 [1.042-1.101] | 2.48E-01 | 2.91E-01 |
| **rs3024493** | *MAPKAPK2, IL10, IL19, IL20* | 1 | C/A | 0.855 | 0.856 | 0.839 | 1.088 [1.050-1.125] | 2.73E-01 | 7.29E-01 |
| **rs4948088** | *COBL* | 7 | C/A | 0.965 | 0.962 | 0.958 | 1.158 [1.087-1.229] | 3.11E-01 | 8.67E-01 |
| **rs11571316** | *CTLA4, ICOS* | 2 | G/A | 0.602 | 0.623 | 0.588 | 1.060 [1.032-1.088] | 3.14E-01 | 2.43E-01 |
| **rs7111341** | *INS,IGF2, INS-IGF2,TH* | 11 | C/T | 0.757 | 0.75 | 0.73 | 1.063 [1.032-1.094] | 3.35E-01 | 6.90E-01 |
| **rs72727394** | *RASGRP1, C15orf53* | 15 | T/C | 0.195 | 0.223 | 0.185 | 1.071 [1.036-1.107] | 3.39E-01 | 5.47E-02 |
| **rs6691977** | *KIF14, DDX59, CAMSAP2, GPR25, C1orf106* | 1 | C/T | 0.213 | 0.219 | 0.207 | 1.065 [1.032-1.098] | 3.56E-01 | 5.00E-01 |
| **rs1456988** | *C14orf64* | 14 | G/T | 0.276 | 0.301 | 0.26 | 1.060 [1.029-1.091] | 3.57E-01 | 3.37E-01 |
| **rs1465788** | *RAD51B, ZFP36L1* | 14 | C/T | 0.735 | 0.733 | 0.728 | 1.059 [1.027-1.090] | 3.70E-01 | 8.25E-01 |
| **rs1615504** | *DOK6, CD226* | 18 | T/C | 0.492 | 0.504 | 0.473 | 1.051 [1.023-1.080] | 3.90E-01 | 3.12E-01 |
| **rs7221109** | *CCR7, SMARCE1, KRT222, KRT222, KRT24* | 17 | C/T | 0.621 | 0.687 | 0.632 | 0.954 [0.925-0.983] | 4.23E-01 | 6.54E-04 |
| **rs12148472** | *CHRNB4, ADAMTS7, MORF4L1, CTSH, RASGRF1* | 15 | T/C | 0.879 | 0.899 | 0.884 | 0.935 [0.893-0.978] | 4.40E-01 | 1.64E-02 |
| **rs1738074** | *RSPH3, TAGAP* | 6 | C/T | 0.607 | 0.568 | 0.585 | 1.045 [1.017-1.073] | 4.43E-01 | 2.67E-02 |
| **rs6920220** | *TNFAIP3* | 6 | A/G | 0.22 | 0.248 | 0.205 | 1.053 [1.019-1.087] | 4.53E-01 | 1.21E-01 |
| **rs1990760** | *GCG, FAP, IFIH1, GCA, KCNH7* | 2 | T/C | 0.633 | 0.651 | 0.618 | 1.043 [1.015-1.071] | 4.59E-01 | 6.66E-01 |
| **rs2611215** | *TMEM192, KLHL2, MSMO1, CPE, TLL1* | 4 | A/G | 0.175 | 0.173 | 0.165 | 1.054 [1.018-1.091] | 4.81E-01 | 7.07E-01 |
| **rs61839660** | *IL2RA, RBM17, PFKFB3* | 10 | C/T | 0.91 | 0.923 | 0.899 | 1.064 [1.017-1.111] | 5.19E-01 | 2.09E-03 |
| **rs7090530** | *IL2RA, RBM17, PFKFB3* | 10 | A/C | 0.605 | 0.645 | 0.611 | 0.964 [0.936-0.993] | 5.34E-01 | 1.12E-02 |
| **rs2281808** | *SIRPD, RP4-576H24.4 (ENSG00000260861), SIRPB1, SIRPG* | 20 | C/T | 0.677 | 0.666 | 0.659 | 1.037 [1.007-1.067] | 5.47E-01 | 5.34E-01 |
| **rs722988** | *ITGB1, NRP1* | 10 | C/T | 0.391 | 0.401 | 0.387 | 1.035 [1.006-1.063] | 5.55E-01 | 6.23E-01 |
| **rs113010081** | *CCR5* | 3 | T/C | 0.887 | 0.901 | 0.882 | 1.052 [1.008-1.097] | 5.77E-01 | 2.05E-01 |
| **rs5753037** | *NF2, CABP7, ZMAT5, UQCR10, ASCC2, MTMR3, HORMAD2, LIF, OSM* | 22 | T/C | 0.381 | 0.415 | 0.375 | 1.032 [1.004-1.060] | 5.83E-01 | 4.47E-01 |
| **rs2304256** | *ICAM1, ICAM4, ICAM5, ZGLP1, FDX1L (ENSG00000167807), FDX1L, CTD-2369P2.12 (ENSG00000267303), RAVER1, ICAM3, TYK2, CDC37, PDE4A, KEAP1, S1PR5* | 19 | C/A | 0.729 | 0.73 | 0.722 | 1.033 [1.003-1.064] | 5.98E-01 | 8.94E-01 |
| **rs11203203** | *UBASH3A* | 21 | A/G | 0.389 | 0.401 | 0.371 | 1.031 [1.002-1.059] | 6.01E-01 | 3.78E-01 |
| **rs705705** | *IKZF4,PMEL, CDK2, RAB5B, SUOX, RPS26, ERBB3, RP11-603J24.9 (ENSG00000257411), PA2G4, RPL41, ZC3H10, ESYT1, MYL6B, MYL6, SMARCC2, RNF41, NABP2, SLC39A5, ANKRD52, COQ10A, CS, RP11-T977G19.10 (ENSG00000144785), CNPY2, PAN2, IL23A, STAT2, APOF* | 12 | C/G | 0.341 | 0.386 | 0.331 | 1.031 [1.002-1.061] | 6.12E-01 | 1.82E-01 |
| **rs478222** | *DNMT3A,CENPO, ADCY3, DNAJC27, EFR3B, POMC* | 2 | A/T | 0.564 | 0.628 | 0.581 | 0.971 [0.943-1.000] | 6.17E-01 | 5.05E-02 |
| **rs11171710** | *IKZF4,PMEL, CDK2, RAB5B, SUOX, RPS26, ERBB3, RP11-603J24.9 (ENSG00000257411), PA2G4, RPL41, ZC3H10, ESYT1, MYL6B, MYL6, SMARCC2, RNF41, NABP2, SLC39A5, ANKRD52, COQ10A, CS, RP11-977G19.10 (ENSG00000144785), CNPY2, PAN2, IL23A, STAT2, APOF* | 12 | A/G | 0.458 | 0.397 | 0.447 | 1.025 [0.998-1.053] | 6.58E-01 | 1.90E-02 |
| **rs7202877** | *CTRB2, CTRB1, BCAR1, CFDP1, RP11-77K12.1 (ENSG00000261717), TMEM170A, CHST6* | 16 | G/T | 0.111 | 0.115 | 0.106 | 1.038 [0.995-1.082] | 6.72E-01 | 2.50E-01 |
| **rs11258747** | *PRKCQ* | 10 | G/T | 0.77 | 0.761 | 0.77 | 1.028 [0.996-1.061] | 6.73E-01 | 6.77E-01 |
| **rs12708716** | *CLEC16A,CIITA, DEXI, RMI2, SOCS1, TNP2, PRM3, PRM2, PRM1, CTD-3088G3.8* | 16 | A/G | 0.637 | 0.702 | 0.633 | 1.024 [0.996-1.052] | 6.84E-01 | 5.55E-03 |
| **rs10795791** | *IL2RA, RBM17* | 10 | G/A | 0.434 | 0.456 | 0.418 | 1.022 [0.994-1.049] | 7.06E-01 | 4.83E-02 |
| **rs3825932** | *CHRNB4, ADAMTS7, MORF4L1, CTSH, RASGRF1* | 15 | T/C | 0.311 | 0.29 | 0.309 | 1.023 [0.993-1.053] | 7.06E-01 | 2.23E-01 |
| **rs10517086** | *SLC34A2, SEL1L3, SMIM20, RBPJ, CCKAR, TBC1D19* | 4 | A/G | 0.303 | 0.326 | 0.305 | 1.021 [0.991-1.050] | 7.33E-01 | 4.80E-01 |
| **rs10492166** | *CD69* | 12 | G/A | 0.508 | 0.549 | 0.506 | 1.019 [0.991-1.046] | 7.40E-01 | 2.10E-02 |
| **rs9924471** | *SBK1, NPIPB6, EIF3CL, NPIPB7, CLN3, CLN3, APOBR, IL27, NUPR1, CCDC101, SULT1A2, SULT1A1, NPIPB8, EIF3C, NPIPB9, ATXN2L, TUFM, SH2B1, ATP2A1, RABEP2, CD19, NFATC2IP, SPNS1, LAT* | 16 | A/G | 0.18 | 0.18 | 0.176 | 1.019 [0.983-1.055] | 7.97E-01 | 7.30E-01 |
| **rs193778** | *CLEC16A,CIITA, DEXI, RMI2, SOCS1, TNP2, PRM3, PRM2, PRM1, CTD-3088G3.8* | 16 | G/A | 0.239 | 0.284 | 0.244 | 1.015 [0.983-1.047] | 8.17E-01 | 5.64E-02 |
| **rs11170466** | *ITGB7* | 12 | T/C | 0.06 | 0.056 | 0.058 | 0.978 [0.921-1.035] | 8.46E-01 | 4.86E-01 |
| **rs924043** | *WDR27, C6orf120, PHF10, TCTE3, ERMARD, DLL1, FAM120B, PSMB1, TBP, PDCD2* | 6 | C/T | 0.852 | 0.867 | 0.856 | 0.989 [0.949-1.029] | 8.94E-01 | 3.16E-01 |
| **rs12453507** | *FBXL20, MED1, CDK12, NEUROD2, PPP1R1B, STARD3, TCAP, PNMT, PGAP3, ERBB2, MIEN1, GRB7, IKZF3, ZPBP2, GSDMB, ORMDL3, LRRC3C, GSDMA, PSMD3, CSF3, MED24, THRA* | 17 | G/C | 0.524 | 0.541 | 0.522 | 1.005 [0.977-1.034] | 9.28E-01 | 8.88E-01 |
| **rs10272724** | *IKZF1, FIGNL1, DDC, GRB10* | 7 | T/C | 0.73 | 0.749 | 0.733 | 1.000 [0.969-1.031] | 9.99E-01 | 6.14E-02 |

**Table S3: LADA cases positive for GADA only (n= 669) vs T1D (BMDCS controls)**

| **RSID** | **Locus** | **Chr** | **T1D Alleles Risk/Other** | **Risk Allele Frequency** | | | **LADA Odds Ratio** | **LADA *P*-value** | **LADA vs T1D *P*-value** |
| --- | --- | --- | --- | --- | --- | --- | --- | --- | --- |
|  |  |  |  | **LADA** | **T1D** | **Control** |  |  |  |
| **rs9272346** | MHC | 6 | A/G | 0.651 | 0.818 | 0.579 | 1.296 [1.266-1.326] | 6.84E-05 | 1.99E-24 |
| **rs17696736** | *SH2B3, NAA25, CUX2, FAM109A,, ATXN2, BRAP, ACAD10, RP11-162P23.2 (ENSG00000257767), ALDH2, MAPKAPK5, TMEM116, ERP29, TRAFD1, HECTD4, RPL6, PTPN11, RPH3A* | 12 | G/A | 0.503 | 0.503 | 0.44 | 1.226 [1.197-1.256] | 1.23E-03 | 0.99 |
| **rs6679677** | *PTPN22, PHTF1* | 1 | A/C | 0.131 | 0.170 | 0.093 | 1.383 [1.336-1.429] | 1.27E-03 | 0.01 |
| **rs9585056** | *UBAC2, GPR18, GPR183, TM9SF2* | 13 | C/T | 0.256 | 0.266 | 0.22 | 1.244 [1.209-1.279] | 3.48E-03 | 0.25 |
| **rs597325** | *BACH2* | 6 | G/A | 0.646 | 0.647 | 0.583 | 1.207 [1.177-1.237] | 3.67E-03 | 0.56 |
| **rs11954020** | *IL7R* | 5 | G/C | 0.444 | 0.415 | 0.387 | 1.191 [1.160-1.221] | 0.01 | 0.17 |
| **rs689** | *INS,IGF2, INS-IGF2,TH* | 11 | T/A | 0.782 | 0.741 | 0.73 | 1.208 [1.175-1.241] | 0.01 | 0.01 |
| **rs229541** | *IL2RB, C1QTNF6, SSTR3, RAC2* | 22 | A/G | 0.446 | 0.468 | 0.412 | 1.169 [1.138-1.199] | 0.02 | 0.14 |
| **rs4505848** | *KIAA1109, ADAD1, IL2, IL21* | 4 | G/A | 0.363 | 0.363 | 0.321 | 1.170 [1.139-1.201] | 0.02 | 0.79 |
| **rs1538171** | *CENPW* | 6 | G/C | 0.518 | 0.475 | 0.472 | 1.156 [1.126-1.186] | 0.02 | 0.15 |
| **rs7804356** | *SKAP2, C7orf71, HOXA1, HOXA2, HOXA3, HOXA4, HOXA5, HOXA6, HOXA7, HOXA9* | 7 | T/C | 0.781 | 0.768 | 0.751 | 1.166 [1.132-1.201] | 0.04 | 0.31 |
| **rs402072** | *DACT3, PRKD2, STRN4, FKRP, SLC1A5, AC008622.1 (ENSG00000280050)* | 19 | T/C | 0.861 | 0.862 | 0.841 | 1.185 [1.143-1.226] | 0.06 | 0.59 |
| **rs601338** | *FUT2,SULT2B1, FAM83E, SPACA4, RPL18, SPHK2, DBP, CA11, NTN5, MAMSTR, RASIP1, IZUMO1, FUT1, FGF21* | 19 | A/G | 0.446 | 0.456 | 0.493 | 0.888 [0.858-0.918] | 0.06 | 0.95 |
| **rs11755527** | *BACH2* | 6 | G/C | 0.474 | 0.5 | 0.43 | 1.119 [1.090-1.148] | 0.07 | 0.14 |
| **rs10877012** | *CYP27B1* | 12 | T/G | 0.344 | 0.313 | 0.311 | 1.120 [1.089-1.152] | 0.09 | 0.17 |
| **rs12908309** | *RASGRP1* | 15 | G/A | 0.779 | 0.78 | 0.76 | 1.137 [1.100-1.175] | 0.11 | 0.43 |
| **rs602662** | *FUT2,SULT2B1, FAM83E, SPACA4, RPL18, SPHK2, DBP, CA11, NTN5, MAMSTR, RASIP1, IZUMO1, FUT1, FGF21* | 19 | G/A | 0.528 | 0.518 | 0.487 | 1.104 [1.074-1.133] | 0.12 | 0.74 |
| **rs2542151** | *PTPN2* | 18 | G/T | 0.149 | 0.202 | 0.166 | 0.887 [0.847-0.927] | 0.16 | 1.20E-03 |
| **rs3024493** | *MAPKAPK2, IL10, IL19, IL20* | 1 | C/A | 0.857 | 0.856 | 0.839 | 1.118 [1.077-1.158] | 0.20 | 0.84 |
| **rs7928968** | *INS,IGF2, INS-IGF2,TH* | 11 | T/A | 0.251 | 0.267 | 0.232 | 1.112 [1.073-1.152] | 0.21 | 0.22 |
| **rs941576** | *DLK1* | 14 | A/G | 0.574 | 0.602 | 0.56 | 1.085 [1.055-1.116] | 0.21 | 0.39 |
| **rs2611215** | *TMEM192, KLHL2, MSMO1, CPE, TLL1* | 4 | A/G | 0.187 | 0.173 | 0.165 | 1.110 [1.070-1.149] | 0.22 | 0.42 |
| **rs12148472** | *CHRNB4, ADAMTS7, MORF4L1, CTSH, RASGRF1* | 15 | T/C | 0.873 | 0.899 | 0.884 | 0.887 [0.842-0.933] | 0.22 | 2.36E-03 |
| **rs4763879** | *CD69, KLRB1, CLEC2D, CLECL1* | 12 | A/G | 0.394 | 0.392 | 0.376 | 1.078 [1.048-1.108] | 0.25 | 0.81 |
| **rs4948088** | *COBL* | 7 | C/A | 0.967 | 0.962 | 0.958 | 1.206 [1.130-1.283] | 0.25 | 0.83 |
| **rs7090530** | *IL2RA, RBM17, PFKFB3* | 10 | A/C | 0.591 | 0.645 | 0.611 | 0.927 [0.897-0.958] | 0.26 | 2.88E-03 |
| **rs72928038** | *BACH2* | 6 | A/G | 0.184 | 0.21 | 0.168 | 1.110 [1.066-1.153] | 0.27 | 3.33E-03 |
| **rs6476839** | *GLIS3* | 9 | T/A | 0.44 | 0.435 | 0.413 | 1.070 [1.040-1.101] | 0.30 | 0.94 |
| **rs10509540** | *RNLS* | 10 | T/C | 0.738 | 0.75 | 0.716 | 1.073 [1.041-1.105] | 0.31 | 0.63 |
| **rs5753037** | *NF2, CABP7, ZMAT5, UQCR10, ASCC2, MTMR3, HORMAD2, LIF, OSM* | 22 | T/C | 0.386 | 0.415 | 0.375 | 1.063 [1.033-1.093] | 0.34 | 0.82 |
| **rs1456988** | *C14orf64* | 14 | G/T | 0.274 | 0.301 | 0.26 | 1.067 [1.033-1.100] | 0.37 | 0.24 |
| **rs9653442** | *AFF3* | 2 | C/T | 0.472 | 0.502 | 0.454 | 1.057 [1.028-1.087] | 0.38 | 0.11 |
| **rs6920220** | *TNFAIP3* | 6 | A/G | 0.22 | 0.248 | 0.205 | 1.061 [1.024-1.097] | 0.45 | 0.12 |
| **rs11171710** | *IKZF4,PMEL, CDK2, RAB5B, SUOX, RPS26, ERBB3, RP11-603J24.9 (ENSG00000257411), PA2G4, RPL41, ZC3H10, ESYT1, MYL6B, MYL6, SMARCC2, RNF41, NABP2, SLC39A5, ANKRD52, COQ10A, CS, RP11-977G19.10 (ENSG00000144785), CNPY2, PAN2, IL23A, STAT2, APOF* | 12 | A/G | 0.466 | 0.397 | 0.447 | 1.049 [1.019-1.079] | 0.45 | 4.31E-03 |
| **rs4849135** | *ACOXL,LINC00116 (ENSG00000175701), LIMS3 (ENSG00000257207), LIMS3L, RGPD6, BUB1, ACOXL* | 2 | G/T | 0.724 | 0.727 | 0.717 | 1.054 [1.021-1.087] | 0.46 | 1.00 |
| **rs694739** | *BAD,MACROD1, FLRT1, STIP1, FERMT3, TRPT1, NUDT22, DNAJC4, VEGFB, FKBP2, PPP1R14B, PLCB3, GPR137, KCNK4, TEX40, ESRRA, TRMT112, PRDX5, CCDC88B, RPS6KA4* | 11 | A/G | 0.626 | 0.639 | 0.608 | 1.049 [1.018-1.079] | 0.47 | 0.32 |
| **rs1465788** | *RAD51B, ZFP36L1* | 14 | C/T | 0.734 | 0.733 | 0.728 | 1.052 [1.019-1.085] | 0.48 | 0.96 |
| **rs10492166** | *CD69* | 12 | G/A | 0.515 | 0.549 | 0.506 | 1.044 [1.015-1.074] | 0.49 | 0.13 |
| **rs722988** | *ITGB1, NRP1* | 10 | C/T | 0.396 | 0.401 | 0.387 | 1.041 [1.010-1.072] | 0.54 | 0.75 |
| **rs11258747** | *PRKCQ* | 10 | G/T | 0.774 | 0.761 | 0.77 | 1.042 [1.007-1.077] | 0.58 | 0.47 |
| **rs924043** | *WDR27, C6orf120, PHF10, TCTE3, ERMARD, DLL1, FAM120B, PSMB1, TBP, PDCD2* | 6 | C/T | 0.845 | 0.867 | 0.856 | 0.951 [0.908-0.994] | 0.59 | 0.13 |
| **rs72727394** | *RASGRP1, C15orf53* | 15 | T/C | 0.194 | 0.223 | 0.185 | 1.045 [1.007-1.083] | 0.59 | 0.05 |
| **rs2281808** | *SIRPD, RP4-576H24.4 (ENSG00000260861), SIRPB1, SIRPG* | 20 | C/T | 0.656 | 0.666 | 0.659 | 0.965 [0.934-0.997] | 0.60 | 0.66 |
| **rs7202877** | *CTRB2, CTRB1, BCAR1, CFDP1, RP11-77K12.1 (ENSG00000261717), TMEM170A, CHST6* | 16 | G/T | 0.114 | 0.115 | 0.106 | 1.053 [1.006-1.100] | 0.61 | 0.31 |
| **rs7111341** | *INS,IGF2, INS-IGF2,TH* | 11 | C/T | 0.731 | 0.75 | 0.73 | 0.970 [0.937-1.003] | 0.67 | 0.23 |
| **rs113010081** | *CCR5* | 3 | T/C | 0.888 | 0.901 | 0.882 | 1.044 [0.996-1.092] | 0.68 | 0.15 |
| **rs1990760** | *GCG, FAP, IFIH1, GCA, KCNH7* | 2 | T/C | 0.629 | 0.651 | 0.618 | 1.027 [0.997-1.057] | 0.68 | 0.67 |
| **rs6691977** | *KIF14, DDX59, CAMSAP2, GPR25, C1orf106* | 1 | C/T | 0.208 | 0.219 | 0.207 | 1.030 [0.994-1.066] | 0.71 | 0.31 |
| **rs10795791** | *IL2RA, RBM17* | 10 | G/A | 0.418 | 0.456 | 0.418 | 0.976 [0.946-1.006] | 0.71 | 0.01 |
| **rs1738074** | *RSPH3, TAGAP* | 6 | C/T | 0.601 | 0.568 | 0.585 | 1.024 [0.993-1.054] | 0.72 | 0.08 |
| **rs1615504** | *DOK6, CD226* | 18 | T/C | 0.488 | 0.504 | 0.473 | 1.023 [0.993-1.054] | 0.73 | 0.22 |
| **rs61839660** | *IL2RA, RBM17, PFKFB3* | 10 | C/T | 0.905 | 0.923 | 0.899 | 1.037 [0.987-1.087] | 0.74 | 3.47E-03 |
| **rs193778** | *CLEC16A,CIITA, DEXI, RMI2, SOCS1, TNP2, PRM3, PRM2, PRM1, CTD-3088G3.8* | 16 | G/A | 0.236 | 0.284 | 0.244 | 1.023 [0.989-1.058] | 0.76 | 0.11 |
| **rs10272724** | *IKZF1, FIGNL1, DDC, GRB10* | 7 | T/C | 0.726 | 0.749 | 0.733 | 0.979 [0.946-1.013] | 0.77 | 0.06 |
| **rs911263** | *RAD51B, ZFP36L1* | 14 | T/C | 0.69 | 0.74 | 0.691 | 1.016 [0.984-1.048] | 0.82 | 0.05 |
| **rs3825932** | *CHRNB4, ADAMTS7, MORF4L1, CTSH, RASGRF1* | 15 | T/C | 0.309 | 0.29 | 0.309 | 1.016 [0.984-1.049] | 0.82 | 0.24 |
| **rs11203203** | *UBASH3A* | 21 | A/G | 0.385 | 0.401 | 0.371 | 1.013 [0.983-1.044] | 0.84 | 0.38 |
| **rs7221109** | *CCR7, SMARCE1, KRT222, KRT222, KRT24* | 17 | C/T | 0.624 | 0.687 | 0.632 | 0.989 [0.958-1.021] | 0.87 | 4.39E-03 |
| **rs2304256** | *ICAM1, ICAM4, ICAM5, ZGLP1, FDX1L (ENSG00000167807), FDX1L, CTD-2369P2.12 (ENSG00000267303), RAVER1, ICAM3, TYK2, CDC37, PDE4A, KEAP1, S1PR5* | 19 | C/A | 0.724 | 0.73 | 0.722 | 1.009 [0.976-1.041] | 0.90 | 0.74 |
| **rs12453507** | *FBXL20, MED1, CDK12, NEUROD2, PPP1R1B, STARD3, TCAP, PNMT, PGAP3, ERBB2, MIEN1, GRB7, IKZF3, ZPBP2, GSDMB, ORMDL3, LRRC3C, GSDMA, PSMD3, CSF3, MED24, THRA* | 17 | G/C | 0.517 | 0.541 | 0.522 | 0.992 [0.962-1.023] | 0.91 | 0.73 |
| **rs9924471** | *SBK1, NPIPB6, EIF3CL, NPIPB7, CLN3, CLN3, APOBR, IL27, NUPR1, CCDC101, SULT1A2, SULT1A1, NPIPB8, EIF3C, NPIPB9, ATXN2L, TUFM, SH2B1, ATP2A1, RABEP2, CD19, NFATC2IP, SPNS1, LAT* | 16 | A/G | 0.174 | 0.18 | 0.824 | 0.992 [0.953-1.031] | 0.92 | 0.48 |
| **rs705705** | *IKZF4,PMEL, CDK2, RAB5B, SUOX, RPS26, ERBB3, RP11-603J24.9 (ENSG00000257411), PA2G4, RPL41, ZC3H10, ESYT1, MYL6B, MYL6, SMARCC2, RNF41, NABP2, SLC39A5, ANKRD52, COQ10A, CS, RP11-977G19.10 (ENSG00000144785), CNPY2, PAN2, IL23A, STAT2, APOF* | 12 | C/G | 0.331 | 0.386 | 0.331 | 0.994 [0.962-1.026] | 0.93 | 0.09 |
| **rs10517086** | *SLC34A2, SEL1L3, SMIM20, RBPJ, CCKAR, TBC1D19* | 4 | A/G | 0.3 | 0.326 | 0.305 | 1.006 [0.974-1.038] | 0.93 | 0.20 |
| **rs12708716** | *CLEC16A,CIITA, DEXI, RMI2, SOCS1, TNP2, PRM3, PRM2, PRM1, CTD-3088G3.8* | 16 | A/G | 0.632 | 0.702 | 0.633 | 1.004 [0.973-1.034] | 0.96 | 0.01 |
| **rs11571316** | *CTLA4, ICOS* | 2 | G/A | 0.587 | 0.623 | 0.588 | 1.002 [0.972-1.032] | 0.97 | 0.10 |
| **rs478222** | *DNMT3A,CENPO, ADCY3, DNAJC27, EFR3B, POMC* | 2 | A/T | 0.574 | 0.628 | 0.581 | 1.002 [0.971-1.033] | 0.98 | 0.11 |
| **rs11170466** | *ITGB7* | 12 | T/C | 0.061 | 0.056 | 0.058 | 0.998 [0.936-1.060] | 0.99 | 0.38 |

**Table S4: LADA cases positive for GADA and IA2A (n= 309) vs T1D (BMDCS controls)**

| **RSID** | **Locus** | **Chr** | **T1D Alleles Risk/Other** | **Risk Allele Frequency** | | | **LADA Odds Ratio** | **LADA *P*-value** | **LADA vs T1D *P*-value** |
| --- | --- | --- | --- | --- | --- | --- | --- | --- | --- |
|  |  |  |  | **LADA** | **T1D** | **Control** |  |  |  |
| **rs9272346** | MHC | 6 | A/G | 0.763 | 0.818 | 0.579 | 1.983 [1.954-2.012] | 1.20E-15 | 4.01E-03 |
| **rs6679677** | *PTPN22, PHTF1* | 1 | A/C | 0.17 | 0.17 | 0.093 | 1.864 [1.819-1.909] | 2.19E-06 | 6.03E-01 |
| **rs17696736** | *SH2B3, NAA25, CUX2, FAM109A,, ATXN2, BRAP, ACAD10, RP11-162P23.2 (ENSG00000257767), ALDH2, MAPKAPK5, TMEM116, ERP29, TRAFD1, HECTD4, RPL6, PTPN11, RPH3A* | 12 | G/A | 0.542 | 0.503 | 0.44 | 1.481 [1.452-1.511] | 5.93E-06 | 1.80E-01 |
| **rs689** | *INS,IGF2, INS-IGF2,TH* | 11 | T/A | 0.824 | 0.741 | 0.73 | 1.440 [1.407-1.474] | 1.90E-04 | 1.68E-06 |
| **rs7111341** | *INS,IGF2, INS-IGF2,TH* | 11 | C/T | 0.812 | 0.75 | 0.73 | 1.360 [1.327-1.394] | 1.82E-03 | 2.39E-04 |
| **rs7804356** | *SKAP2, C7orf71, HOXA1, HOXA2, HOXA3, HOXA4, HOXA5, HOXA6, HOXA7, HOXA9* | 7 | T/C | 0.814 | 0.768 | 0.751 | 1.354 [1.320-1.388] | 2.27E-03 | 1.22E-02 |
| **rs2281808** | *SIRPD, RP4-576H24.4 (ENSG00000260861), SIRPB1, SIRPG* | 20 | C/T | 0.723 | 0.666 | 0.659 | 1.276 [1.244-1.308] | 9.17E-03 | 9.53E-03 |
| **rs694739** | *BAD,MACROD1, FLRT1, STIP1, FERMT3, TRPT1, NUDT22, DNAJC4, VEGFB, FKBP2, PPP1R14B, PLCB3, GPR137, KCNK4, TEX40, ESRRA, TRMT112, PRDX5, CCDC88B, RPS6KA4* | 11 | A/G | 0.672 | 0.639 | 0.608 | 1.253 [1.223-1.283] | 1.06E-02 | 3.07E-01 |
| **rs911263** | *RAD51B, ZFP36L1* | 14 | T/C | 0.739 | 0.74 | 0.691 | 1.265 [1.233-1.297] | 1.09E-02 | 6.27E-01 |
| **rs72928038** | *BACH2* | 6 | A/G | 0.213 | 0.21 | 0.168 | 1.367 [1.324-1.410] | 1.31E-02 | 4.29E-01 |
| **rs9653442** | *AFF3* | 2 | C/T | 0.515 | 0.502 | 0.454 | 1.235 [1.205-1.264] | 1.37E-02 | 5.59E-01 |
| **rs11571316** | *CTLA4, ICOS* | 2 | G/A | 0.634 | 0.623 | 0.588 | 1.238 [1.208-1.268] | 1.48E-02 | 5.03E-01 |
| **rs11755527** | *BACH2* | 6 | G/C | 0.495 | 0.5 | 0.43 | 1.229 [1.200-1.258] | 1.54E-02 | 6.10E-01 |
| **rs7928968** | *INS,IGF2, INS-IGF2,TH* | 11 | T/A | 0.276 | 0.267 | 0.232 | 1.292 [1.253-1.331] | 2.32E-02 | 5.67E-01 |
| **rs597325** | *BACH2* | 6 | G/A | 0.646 | 0.647 | 0.583 | 1.216 [1.186-1.246] | 2.68E-02 | 6.67E-01 |
| **rs10795791** | *IL2RA, RBM17* | 10 | G/A | 0.469 | 0.456 | 0.418 | 1.200 [1.171-1.229] | 3.39E-02 | 6.80E-01 |
| **rs6476839** | *GLIS3* | 9 | T/A | 0.465 | 0.435 | 0.413 | 1.188 [1.158-1.219] | 5.29E-02 | 2.04E-01 |
| **rs4505848** | *KIAA1109, ADAD1, IL2, IL21* | 4 | G/A | 0.354 | 0.363 | 0.321 | 1.161 [1.130-1.192] | 1.01E-01 | 9.74E-01 |
| **rs705705** | *IKZF4,PMEL, CDK2, RAB5B, SUOX, RPS26, ERBB3, RP11-603J24.9 (ENSG00000257411), PA2G4, RPL41, ZC3H10, ESYT1, MYL6B, MYL6, SMARCC2, RNF41, NABP2, SLC39A5, ANKRD52, COQ10A, CS, RP11-977G19.10 (ENSG00000144785), CNPY2, PAN2, IL23A, STAT2, APOF* | 12 | C/G | 0.364 | 0.386 | 0.331 | 1.158 [1.126-1.190] | 1.14E-01 | 7.76E-01 |
| **rs11954020** | *IL7R* | 5 | G/C | 0.424 | 0.415 | 0.387 | 1.149 [1.118-1.179] | 1.18E-01 | 5.20E-01 |
| **rs4763879** | *CD69, KLRB1, CLEC2D, CLECL1* | 12 | A/G | 0.405 | 0.392 | 0.376 | 1.139 [1.108-1.169] | 1.48E-01 | 7.25E-01 |
| **rs61839660** | *IL2RA, RBM17, PFKFB3* | 10 | C/T | 0.921 | 0.923 | 0.899 | 1.223 [1.173-1.273] | 1.66E-01 | 2.66E-01 |
| **rs1738074** | *RSPH3, TAGAP* | 6 | C/T | 0.62 | 0.568 | 0.585 | 1.119 [1.089-1.148] | 1.93E-01 | 1.26E-02 |
| **rs402072** | *DACT3, PRKD2, STRN4, FKRP, SLC1A5, AC008622.1 (ENSG00000280050)* | 19 | T/C | 0.861 | 0.862 | 0.841 | 1.168 [1.127-1.209] | 1.93E-01 | 7.25E-01 |
| **rs10509540** | *RNLS* | 10 | T/C | 0.748 | 0.75 | 0.716 | 1.123 [1.090-1.155] | 2.19E-01 | 9.90E-01 |
| **rs478222** | *DNMT3A,CENPO, ADCY3, DNAJC27, EFR3B, POMC* | 2 | A/T | 0.542 | 0.628 | 0.581 | 0.896 [0.865-0.927] | 2.24E-01 | 1.32E-02 |
| **rs7221109** | *CCR7, SMARCE1, KRT222, KRT222, KRT24* | 17 | C/T | 0.615 | 0.687 | 0.632 | 0.898 [0.867-0.928] | 2.27E-01 | 1.96E-03 |
| **rs4849135** | *ACOXL,LINC00116 (ENSG00000175701), LIMS3 (ENSG00000257207), LIMS3L, RGPD6, BUB1, ACOXL* | 2 | G/T | 0.744 | 0.727 | 0.717 | 1.122 [1.090-1.155] | 2.28E-01 | 6.52E-01 |
| **rs2542151** | *PTPN2* | 18 | G/T | 0.147 | 0.202 | 0.166 | 0.877 [0.838-0.916] | 2.50E-01 | 4.07E-02 |
| **rs1615504** | *DOK6, CD226* | 18 | T/C | 0.499 | 0.504 | 0.473 | 1.106 [1.076-1.136] | 2.50E-01 | 6.01E-01 |
| **rs6691977** | *KIF14, DDX59, CAMSAP2, GPR25, C1orf106* | 1 | C/T | 0.223 | 0.219 | 0.207 | 1.124 [1.089-1.160] | 2.58E-01 | 8.94E-01 |
| **rs9924471** | *SBK1, NPIPB6, EIF3CL, NPIPB7, CLN3, CLN3, APOBR, IL27, NUPR1, CCDC101, SULT1A2, SULT1A1, NPIPB8, EIF3C, NPIPB9, ATXN2L, TUFM, SH2B1, ATP2A1, RABEP2, CD19, NFATC2IP, SPNS1, LAT* | 16 | A/G | 0.193 | 0.18 | 0.176 | 1.133 [1.095-1.172] | 2.65E-01 | 7.40E-01 |
| **rs1990760** | *GCG, FAP, IFIH1, GCA, KCNH7* | 2 | T/C | 0.642 | 0.651 | 0.618 | 1.100 [1.070-1.130] | 2.69E-01 | 7.60E-01 |
| **rs12908309** | *RASGRP1* | 15 | G/A | 0.778 | 0.78 | 0.76 | 1.124 [1.087-1.160] | 2.69E-01 | 3.31E-01 |
| **rs1538171** | *CENPW* | 6 | G/C | 0.505 | 0.475 | 0.472 | 1.099 [1.069-1.128] | 2.76E-01 | 4.97E-01 |
| **rs2304256** | *ICAM1, ICAM4, ICAM5, ZGLP1, FDX1L (ENSG00000167807), FDX1L, CTD-2369P2.12 (ENSG00000267303), RAVER1, ICAM3, TYK2, CDC37, PDE4A, KEAP1, S1PR5* | 19 | C/A | 0.739 | 0.73 | 0.722 | 1.107 [1.074-1.140] | 2.92E-01 | 3.85E-01 |
| **rs12708716** | *CLEC16A,CIITA, DEXI, RMI2, SOCS1, TNP2, PRM3, PRM2, PRM1, CTD-3088G3.8* | 16 | A/G | 0.647 | 0.702 | 0.633 | 1.093 [1.063-1.124] | 3.15E-01 | 9.47E-02 |
| **rs7090530** | *IL2RA, RBM17, PFKFB3* | 10 | A/C | 0.636 | 0.645 | 0.611 | 1.089 [1.059-1.119] | 3.32E-01 | 9.76E-01 |
| **rs1456988** | *C14orf64* | 14 | G/T | 0.278 | 0.301 | 0.26 | 1.097 [1.065-1.130] | 3.32E-01 | 7.46E-01 |
| **rs2611215** | *TMEM192, KLHL2, MSMO1, CPE, TLL1* | 4 | A/G | 0.149 | 0.173 | 0.165 | 0.898 [0.858-0.938] | 3.57E-01 | 2.38E-01 |
| **rs72727394** | *RASGRP1, C15orf53* | 15 | T/C | 0.198 | 0.223 | 0.185 | 1.103 [1.065-1.140] | 3.75E-01 | 1.82E-01 |
| **rs11203203** | *UBASH3A* | 21 | A/G | 0.396 | 0.401 | 0.371 | 1.079 [1.049-1.109] | 3.82E-01 | 9.08E-01 |
| **rs941576** | *DLK1* | 14 | A/G | 0.574 | 0.602 | 0.56 | 1.079 [1.049-1.109] | 3.84E-01 | 6.79E-01 |
| **rs601338** | *FUT2,SULT2B1, FAM83E, SPACA4, RPL18, SPHK2, DBP, CA11, NTN5, MAMSTR, RASIP1, IZUMO1, FUT1, FGF21* | 19 | A/G | 0.461 | 0.456 | 0.493 | 0.935 [0.906-0.964] | 4.30E-01 | 7.86E-01 |
| **rs3024493** | *MAPKAPK2, IL10, IL19, IL20* | 1 | C/A | 0.85 | 0.856 | 0.839 | 1.087 [1.047-1.126] | 4.73E-01 | 7.16E-01 |
| **rs924043** | *WDR27, C6orf120, PHF10, TCTE3, ERMARD, DLL1, FAM120B, PSMB1, TBP, PDCD2* | 6 | C/T | 0.866 | 0.867 | 0.856 | 1.084 [1.041-1.127] | 5.19E-01 | 7.56E-01 |
| **rs229541** | *IL2RB, C1QTNF6, SSTR3, RAC2* | 22 | A/G | 0.414 | 0.468 | 0.412 | 1.057 [1.026-1.089] | 5.39E-01 | 2.07E-02 |
| **rs12148472** | *CHRNB4, ADAMTS7, MORF4L1, CTSH, RASGRF1* | 15 | T/C | 0.893 | 0.899 | 0.884 | 1.085 [1.038-1.131] | 5.48E-01 | 8.32E-01 |
| **rs10517086** | *SLC34A2, SEL1L3, SMIM20, RBPJ, CCKAR, TBC1D19* | 4 | A/G | 0.309 | 0.326 | 0.305 | 1.054 [1.023-1.085] | 5.61E-01 | 9.95E-01 |
| **rs1465788** | *RAD51B, ZFP36L1* | 14 | C/T | 0.736 | 0.733 | 0.728 | 1.056 [1.023-1.090] | 5.76E-01 | 9.36E-01 |
| **rs11171710** | *IKZF4,PMEL, CDK2, RAB5B, SUOX, RPS26, ERBB3, RP11-603J24.9 (ENSG00000257411), PA2G4, RPL41, ZC3H10, ESYT1, MYL6B, MYL6, SMARCC2, RNF41, NABP2, SLC39A5, ANKRD52, COQ10A, CS, RP11-977G19.10 (ENSG00000144785), CNPY2, PAN2, IL23A, STAT2, APOF* | 12 | A/G | 0.44 | 0.397 | 0.447 | 0.953 [0.923-0.983] | 5.78E-01 | 3.13E-01 |
| **rs6920220** | *TNFAIP3* | 6 | A/G | 0.218 | 0.248 | 0.205 | 1.059 [1.023-1.095] | 5.85E-01 | 2.27E-01 |
| **rs602662** | *FUT2,SULT2B1, FAM83E, SPACA4, RPL18, SPHK2, DBP, CA11, NTN5, MAMSTR, RASIP1, IZUMO1, FUT1, FGF21* | 19 | G/A | 0.513 | 0.518 | 0.487 | 1.045 [1.016-1.074] | 6.04E-01 | 6.69E-01 |
| **rs12453507** | *FBXL20, MED1, CDK12, NEUROD2, PPP1R1B, STARD3, TCAP, PNMT, PGAP3, ERBB2, MIEN1, GRB7, IKZF3, ZPBP2, GSDMB, ORMDL3, LRRC3C, GSDMA, PSMD3, CSF3, MED24, THRA* | 17 | G/C | 0.538 | 0.541 | 0.522 | 1.041 [1.011-1.072] | 6.48E-01 | 7.67E-01 |
| **rs9585056** | *UBAC2, GPR18, GPR183, TM9SF2* | 13 | C/T | 0.227 | 0.266 | 0.22 | 1.045 [1.010-1.080] | 6.68E-01 | 2.18E-02 |
| **rs3825932** | *CHRNB4, ADAMTS7, MORF4L1, CTSH, RASGRF1* | 15 | T/C | 0.314 | 0.29 | 0.309 | 1.041 [1.009-1.074] | 6.70E-01 | 3.32E-01 |
| **rs10272724** | *IKZF1, FIGNL1, DDC, GRB10* | 7 | T/C | 0.738 | 0.749 | 0.733 | 1.025 [0.992-1.058] | 8.00E-01 | 3.56E-01 |
| **rs193778** | *CLEC16A,CIITA, DEXI, RMI2, SOCS1, TNP2, PRM3, PRM2, PRM1, CTD-3088G3.8* | 16 | G/A | 0.244 | 0.284 | 0.244 | 1.024 [0.990-1.059] | 8.10E-01 | 1.62E-01 |
| **rs10492166** | *CD69* | 12 | G/A | 0.49 | 0.549 | 0.506 | 0.981 [0.951-1.010] | 8.20E-01 | 1.82E-02 |
| **rs11170466** | *ITGB7* | 12 | T/C | 0.06 | 0.056 | 0.058 | 0.965 [0.904-1.026] | 8.42E-01 | 9.89E-01 |
| **rs722988** | *ITGB1, NRP1* | 10 | C/T | 0.379 | 0.401 | 0.387 | 1.016 [0.985-1.046] | 8.59E-01 | 5.64E-01 |
| **rs11258747** | *PRKCQ* | 10 | G/T | 0.764 | 0.761 | 0.77 | 0.989 [0.954-1.024] | 9.11E-01 | 8.21E-01 |
| **rs10877012** | *CYP27B1* | 12 | T/G | 0.32 | 0.313 | 0.311 | 1.007 [0.976-1.038] | 9.39E-01 | 5.86E-01 |
| **rs5753037** | *NF2, CABP7, ZMAT5, UQCR10, ASCC2, MTMR3, HORMAD2, LIF, OSM* | 22 | T/C | 0.371 | 0.415 | 0.375 | 0.994 [0.964-1.024] | 9.45E-01 | 2.26E-01 |
| **rs4948088** | *COBL* | 7 | C/A | 0.96 | 0.962 | 0.958 | 1.007 [0.933-1.081] | 9.74E-01 | 3.03E-01 |
| **rs113010081** | *CCR5* | 3 | T/C | 0.886 | 0.901 | 0.882 | 0.998 [0.951-1.046] | 9.91E-01 | 1.85E-01 |
| **rs7202877** | *CTRB2, CTRB1, BCAR1, CFDP1, RP11-77K12.1 (ENSG00000261717), TMEM170A, CHST6* | 16 | G/T | 0.105 | 0.115 | 0.106 | 1.001 [0.954-1.048] | 9.94E-01 | 3.51E-01 |

**Table S5: LADA (n= 978) vs T2D (BMDCS controls)**

| **RSID** | **Locus** | **Chr** | **T2D Alleles Risk/Other** | **Risk Allele Frequency** | | | **LADA Odds Ratio** | **LADA *P*-value** | **LADA vs T2D *P*-value** |
| --- | --- | --- | --- | --- | --- | --- | --- | --- | --- |
|  |  |  |  | **LADA** | **T2D** | **Control** |  |  |  |
| **rs12427353** | *HNF1A* | 12 | G/C | 0.831 | 0.828 | 0.787 | 1.291 [1.256-1.326] | 3.42E-04 | 5.38E-01 |
| **rs849135** | *JAZF1* | 7 | G/A | 0.526 | 0.529 | 0.485 | 1.165 [1.137-1.193] | 7.99E-03 | 8.10E-01 |
| **rs6723108** | *TMEM163* | 2 | T/G | 0.617 | 0.521 | 0.553 | 1.149 [1.121-1.177] | 1.58E-02 | 2.21E-03 |
| **rs11634397** | *ZFAND6* | 15 | G/A | 0.683 | 0.679 | 0.653 | 1.152 [1.123-1.181] | 1.67E-02 | 9.25E-01 |
| **rs8108269** | *GIPR* | 19 | G/T | 0.338 | 0.31 | 0.292 | 1.153 [1.123-1.183] | 1.96E-02 | 1.70E-01 |
| **rs7178572** | *HMG20A* | 15 | G/A | 0.702 | 0.73 | 0.73 | 0.870 [0.839-0.900] | 2.58E-02 | 8.38E-02 |
| **rs12970134** | *MC4R* | 18 | A/G | 0.28 | 0.291 | 0.247 | 1.151 [1.120-1.181] | 2.59E-02 | 5.59E-01 |
| **rs16861329** | *ST64GAL1* | 3 | C/T | 0.882 | 0.872 | 0.861 | 1.186 [1.145-1.226] | 3.92E-02 | 3.61E-01 |
| **rs1111875** | *HHEX/IDE* | 10 | C/T | 0.625 | 0.634 | 0.59 | 1.115 [1.087-1.142] | 5.36E-02 | 3.24E-01 |
| **rs17106184** | *FAF1* | 1 | G/A | 0.91 | 0.918 | 0.895 | 1.191 [1.146-1.237] | 6.00E-02 | 6.55E-01 |
| **rs2943640** | *IRS1* | 2 | C/A | 0.662 | 0.675 | 0.634 | 1.118 [1.089-1.148] | 6.58E-02 | 8.53E-01 |
| **rs163184** | *KCNQ1* | 11 | G/T | 0.497 | 0.49 | 0.463 | 1.104 [1.077-1.132] | 8.09E-02 | 6.43E-01 |
| **rs10923931** | *NOTCH2* | 1 | T/G | 0.117 | 0.117 | 0.106 | 1.161 [1.119-1.204] | 8.48E-02 | 9.67E-01 |
| **rs6813195** | *TMEM154* | 4 | C/T | 0.74 | 0.737 | 0.718 | 1.110 [1.079-1.141] | 1.03E-01 | 6.24E-01 |
| **rs7041847** | *GLIS3* | 9 | A/G | 0.535 | 0.516 | 0.505 | 1.097 [1.068-1.125] | 1.12E-01 | 2.24E-01 |
| **rs5215** | *KCNJ11* | 11 | C/T | 0.382 | 0.382 | 0.362 | 1.097 [1.068-1.125] | 1.15E-01 | 4.90E-01 |
| **rs17168486** | *DGKB* | 7 | T/C | 0.187 | 0.172 | 0.163 | 1.120 [1.085-1.156] | 1.17E-01 | 3.41E-01 |
| **rs4430796** | *HNF1B* | 17 | G/A | 0.512 | 0.479 | 0.484 | 1.092 [1.064-1.121] | 1.26E-01 | 2.51E-02 |
| **rs10811661** | *CDKN2A/B* | 9 | T/C | 0.851 | 0.858 | 0.831 | 1.120 [1.082-1.157] | 1.39E-01 | 6.04E-01 |
| **rs10203174** | *THADA* | 2 | C/T | 0.887 | 0.897 | 0.871 | 1.136 [1.094-1.178] | 1.40E-01 | 9.93E-02 |
| **rs11063069** | *CCND2* | 12 | G/A | 0.21 | 0.223 | 0.221 | 0.911 [0.878-0.944] | 1.65E-01 | 2.69E-01 |
| **rs10886471** | *GRK5* | 10 | T/C | 0.469 | 0.477 | 0.489 | 0.927 [0.899-0.954] | 1.78E-01 | 3.66E-01 |
| **rs11717195** | *ADCY5* | 3 | T/C | 0.783 | 0.777 | 0.77 | 1.096 [1.062-1.130] | 1.84E-01 | 3.07E-01 |
| **rs391300** | *SRR* | 17 | C/T | 0.632 | 0.638 | 0.61 | 1.079 [1.051-1.107] | 1.88E-01 | 7.14E-01 |
| **rs780094** | *GCKR* | 2 | C/T | 0.571 | 0.618 | 0.6 | 0.928 [0.900-0.956] | 1.88E-01 | 3.15E-02 |
| **rs7403531** | *RASGRP1* | 15 | T/C | 0.221 | 0.229 | 0.204 | 1.091 [1.058-1.124] | 1.97E-01 | 3.29E-01 |
| **rs1802295** | *VPS26A* | 10 | T/C | 0.302 | 0.321 | 0.322 | 0.925 [0.895-0.956] | 2.12E-01 | 1.21E-01 |
| **rs17584499** | *PTPRD* | 9 | T/C | 0.212 | 0.17 | 0.193 | 1.084 [1.051-1.118] | 2.35E-01 | 1.54E-03 |
| **rs516946** | *ANK1* | 8 | C/T | 0.767 | 0.782 | 0.754 | 1.080 [1.048-1.112] | 2.42E-01 | 7.69E-02 |
| **rs3786897** | *PEPD* | 19 | A/G | 0.608 | 0.578 | 0.589 | 1.070 [1.041-1.098] | 2.48E-01 | 6.75E-02 |
| **rs2261181** | *HMGA2* | 12 | T/C | 0.115 | 0.109 | 0.101 | 1.111 [1.066-1.155] | 2.54E-01 | 9.26E-01 |
| **rs4275659** | *MPHOSPH9* | 12 | C/T | 0.715 | 0.735 | 0.694 | 1.072 [1.042-1.102] | 2.55E-01 | 8.06E-01 |
| **rs3923113** | *GRB14* | 2 | A/C | 0.646 | 0.656 | 0.632 | 1.062 [1.034-1.091] | 3.03E-01 | 1.76E-01 |
| **rs7955901** | *TSPAN8* | 12 | C/T | 0.461 | 0.487 | 0.438 | 1.058 [1.031-1.086] | 3.11E-01 | 1.29E-01 |
| **rs3802177** | *SLC30A8* | 8 | G/A | 0.684 | 0.706 | 0.699 | 0.941 [0.911-0.971] | 3.23E-01 | 5.96E-02 |
| **rs4458523** | *WFS1* | 4 | G/T | 0.611 | 0.614 | 0.59 | 1.056 [1.028-1.084] | 3.36E-01 | 9.89E-01 |
| **rs7163757** | *C2CD4A* | 15 | C/T | 0.573 | 0.59 | 0.588 | 0.950 [0.923-0.978] | 3.65E-01 | 3.76E-02 |
| **rs6878122** | *ZBED3* | 5 | A/G | 0.718 | 0.658 | 0.706 | 1.051 [1.021-1.081] | 4.20E-01 | 8.81E-04 |
| **rs17791513** | *TLE4* | 9 | A/G | 0.946 | 0.952 | 0.939 | 1.096 [1.037-1.154] | 4.45E-01 | 6.81E-01 |
| **rs459193** | *ANKRD55* | 5 | G/A | 0.753 | 0.771 | 0.741 | 1.050 [1.018-1.082] | 4.55E-01 | 1.43E-01 |
| **rs13233731** | *KLF14* | 7 | G/A | 0.544 | 0.538 | 0.527 | 1.043 [1.015-1.072] | 4.67E-01 | 9.79E-01 |
| **rs7593730** | *RBMS1* | 2 | C/T | 0.793 | 0.811 | 0.796 | 1.052 [1.018-1.085] | 4.68E-01 | 4.54E-01 |
| **rs2075423** | *PROX1* | 1 | G/T | 0.643 | 0.678 | 0.653 | 0.959 [0.930-0.988] | 4.75E-01 | 2.41E-01 |
| **rs10842994** | *KLHDC5* | 12 | C/T | 0.803 | 0.814 | 0.794 | 1.050 [1.016-1.083] | 4.84E-01 | 4.23E-01 |
| **rs1359790** | *SPRY2* | 13 | G/A | 0.746 | 0.741 | 0.736 | 1.043 [1.011-1.075] | 5.16E-01 | 5.07E-01 |
| **rs243088** | *BCL11A* | 2 | T/A | 0.494 | 0.492 | 0.485 | 1.036 [1.008-1.064] | 5.32E-01 | 4.08E-01 |
| **rs10401969** | *CILP2* | 19 | C/T | 0.077 | 0.088 | 0.067 | 1.069 [1.016-1.122] | 5.37E-01 | 7.38E-02 |
| **rs11257655** | *CDC123* | 10 | T/C | 0.194 | 0.236 | 0.213 | 0.962 [0.927-0.997] | 5.85E-01 | 9.13E-02 |
| **rs7756992** | *CDKAL1* | 6 | G/A | 0.306 | 0.316 | 0.289 | 1.033 [1.003-1.062] | 5.96E-01 | 6.09E-01 |
| **rs2796441** | *TLE1* | 9 | G/A | 0.607 | 0.582 | 0.601 | 1.031 [1.003-1.058] | 5.97E-01 | 4.85E-02 |
| **rs7202877** | *BCAR1* | 16 | T/G | 0.889 | 0.914 | 0.894 | 0.963 [0.920-1.007] | 6.72E-01 | 4.14E-02 |
| **rs4812829** | *HNF4A* | 20 | G/A | 0.836 | 0.837 | 0.831 | 1.033 [0.995-1.070] | 6.74E-01 | 3.23E-01 |
| **rs7612463** | *UBE2E2* | 3 | C/A | 0.887 | 0.894 | 0.89 | 0.963 [0.918-1.007] | 6.75E-01 | 1.19E-01 |
| **rs10830963** | *MTNR1B* | 11 | G/C | 0.292 | 0.27 | 0.29 | 0.974 [0.944-1.005] | 6.76E-01 | 2.28E-01 |
| **rs10278336** | *GCK* | 7 | A/G | 0.58 | 0.577 | 0.579 | 1.024 [0.996-1.053] | 6.82E-01 | 3.21E-01 |
| **rs12899811** | *PRC1* | 15 | G/A | 0.324 | 0.315 | 0.306 | 1.024 [0.994-1.053] | 6.98E-01 | 9.54E-01 |
| **rs7903146** | *TCF7L2* | 10 | T/C | 0.295 | 0.376 | 0.298 | 1.023 [0.994-1.053] | 7.02E-01 | 5.21E-06 |
| **rs9936385** | *FTO* | 16 | C/T | 0.4 | 0.454 | 0.394 | 1.022 [0.994-1.050] | 7.09E-01 | 1.80E-03 |
| **rs6795735** | *ADAMTS9* | 3 | C/T | 0.571 | 0.618 | 0.575 | 0.982 [0.955-1.009] | 7.45E-01 | 1.26E-02 |
| **rs9505118** | *SSR1/RREB1* | 6 | A/G | 0.595 | 0.612 | 0.592 | 1.018 [0.989-1.046] | 7.60E-01 | 1.91E-01 |
| **rs831571** | *PSMD6* | 3 | C/T | 0.829 | 0.81 | 0.827 | 1.023 [0.986-1.059] | 7.64E-01 | 1.74E-01 |
| **rs7845219** | *TP53INP1* | 8 | T/C | 0.515 | 0.525 | 0.515 | 0.983 [0.955-1.011] | 7.65E-01 | 3.86E-01 |
| **rs4402960** | *IGF2BP* | 3 | T/G | 0.305 | 0.351 | 0.314 | 0.983 [0.952-1.013] | 7.82E-01 | 1.01E-02 |
| **rs12571751** | *ZMIZ1* | 10 | A/G | 0.544 | 0.583 | 0.549 | 0.988 [0.960-1.015] | 8.27E-01 | 1.02E-02 |
| **rs2334499** | *DUSP8* | 11 | T/C | 0.409 | 0.418 | 0.41 | 1.011 [0.983-1.039] | 8.52E-01 | 1.93E-01 |
| **rs6467136** | *GCC1* | 7 | A/G | 0.479 | 0.472 | 0.477 | 1.008 [0.981-1.036] | 8.85E-01 | 7.75E-01 |
| **rs9470794** | *ZFAND3* | 6 | T/C | 0.908 | 0.911 | 0.908 | 1.014 [0.966-1.061] | 8.89E-01 | 3.93E-01 |
| **rs2028299** | *AP3S2* | 15 | C/A | 0.284 | 0.297 | 0.291 | 0.995 [0.965-1.026] | 9.42E-01 | 7.89E-01 |
| **rs1552224** | *ARAP1 (CENTD2)* | 11 | A/C | 0.851 | 0.849 | 0.85 | 1.005 [0.967-1.043] | 9.45E-01 | 8.96E-01 |
| **rs702634** | *ARL15* | 5 | A/G | 0.306 | 0.289 | 0.299 | 0.997 [0.967-1.027] | 9.56E-01 | 5.92E-01 |
| **rs6808574** | *LPP* | 3 | C/T | 0.621 | 0.604 | 0.613 | 1.003 [0.975-1.031] | 9.60E-01 | 7.07E-01 |

**Table S6: LADA cases positive for GADA only (n= 669) vs T2D (BMDCS controls)**

| **RSID** | **Locus** | **Chr** | **T2D Alleles Risk/Other** | **Risk Allele Frequency** | | | **LADA Odds Ratio** | **LADA *P*-value** | **LADA vs T2D *P*-value** |
| --- | --- | --- | --- | --- | --- | --- | --- | --- | --- |
|  |  |  |  | **LADA** | **T2D** | **Control** |  |  |  |
| **rs1111875** | *HHEX/IDE* | 10 | C/T | 0.643 | 0.634 | 0.59 | 1.205 [1.175-1.234] | 3.63E-03 | 0.75 |
| **rs12970134** | *MC4R* | 18 | A/G | 0.291 | 0.291 | 0.247 | 1.216 [1.183-1.249] | 0.01 | 0.97 |
| **rs12427353** | *HNF1A* | 12 | G/C | 0.818 | 0.828 | 0.787 | 1.224 [1.187-1.260] | 0.01 | 0.72 |
| **rs11634397** | *ZFAND6* | 15 | G/A | 0.69 | 0.679 | 0.653 | 1.184 [1.153-1.215] | 0.01 | 0.80 |
| **rs16861329** | *ST64GAL1* | 3 | C/T | 0.886 | 0.872 | 0.861 | 1.251 [1.208-1.294] | 0.02 | 0.29 |
| **rs849135** | *JAZF1* | 7 | G/A | 0.527 | 0.529 | 0.485 | 1.168 [1.138-1.198] | 0.02 | 0.97 |
| **rs7178572** | *HMG20A* | 15 | G/A | 0.699 | 0.73 | 0.73 | 0.851 [0.818-0.884] | 0.02 | 0.08 |
| **rs4458523** | *WFS1* | 4 | G/T | 0.628 | 0.614 | 0.59 | 1.143 [1.113-1.173] | 0.04 | 0.31 |
| **rs6723108** | *TMEM163* | 2 | T/G | 0.613 | 0.521 | 0.553 | 1.144 [1.114-1.174] | 0.04 | 4.27E-03 |
| **rs3786897** | *PEPD* | 19 | A/G | 0.625 | 0.578 | 0.589 | 1.143 [1.112-1.173] | 0.04 | 0.02 |
| **rs11717195** | *ADCY5* | 3 | T/C | 0.796 | 0.777 | 0.77 | 1.162 [1.126-1.199] | 0.06 | 0.18 |
| **rs8108269** | *GIPR* | 19 | G/T | 0.336 | 0.31 | 0.292 | 1.142 [1.110-1.175] | 0.06 | 0.26 |
| **rs10203174** | *THADA* | 2 | C/T | 0.89 | 0.897 | 0.871 | 1.200 [1.154-1.246] | 0.06 | 0.18 |
| **rs6813195** | *TMEM154* | 4 | C/T | 0.748 | 0.737 | 0.718 | 1.141 [1.107-1.175] | 0.07 | 0.39 |
| **rs10811661** | *CDKN2A/B* | 9 | T/C | 0.86 | 0.858 | 0.831 | 1.170 [1.130-1.211] | 0.07 | 0.64 |
| **rs163184** | *KCNQ1* | 11 | G/T | 0.498 | 0.49 | 0.463 | 1.124 [1.094-1.154] | 0.07 | 0.39 |
| **rs17106184** | *FAF1* | 1 | G/A | 0.913 | 0.918 | 0.895 | 1.207 [1.158-1.256] | 0.07 | 0.57 |
| **rs5215** | *KCNJ11* | 11 | C/T | 0.386 | 0.382 | 0.362 | 1.112 [1.081-1.142] | 0.11 | 0.41 |
| **rs391300** | *SRR* | 17 | C/T | 0.638 | 0.638 | 0.61 | 1.109 [1.078-1.139] | 0.11 | 0.98 |
| **rs4430796** | *HNF1B* | 17 | G/A | 0.517 | 0.479 | 0.484 | 1.108 [1.078-1.138] | 0.12 | 0.03 |
| **rs17584499** | *PTPRD* | 9 | T/C | 0.217 | 0.17 | 0.193 | 1.130 [1.094-1.167] | 0.12 | 9.54E-04 |
| **rs10923931** | *NOTCH2* | 1 | T/G | 0.12 | 0.117 | 0.106 | 1.164 [1.118-1.209] | 0.12 | 0.91 |
| **rs2943640** | *IRS1* | 2 | C/A | 0.656 | 0.675 | 0.634 | 1.108 [1.076-1.140] | 0.13 | 0.47 |
| **rs516946** | *ANK1* | 8 | C/T | 0.775 | 0.782 | 0.754 | 1.116 [1.082-1.151] | 0.14 | 0.22 |
| **rs1359790** | *SPRY2* | 13 | G/A | 0.76 | 0.741 | 0.736 | 1.104 [1.070-1.139] | 0.18 | 0.75 |
| **rs10401969** | *CILP2* | 19 | C/T | 0.085 | 0.088 | 0.067 | 1.172 [1.115-1.228] | 0.19 | 0.35 |
| **rs7903146** | *TCF7L2* | 10 | T/C | 0.315 | 0.376 | 0.298 | 1.088 [1.056-1.119] | 0.21 | 5.03E-04 |
| **rs3923113** | *GRB14* | 2 | A/C | 0.65 | 0.656 | 0.632 | 1.081 [1.050-1.112] | 0.24 | 0.36 |
| **rs2261181** | *HMGA2* | 12 | T/C | 0.114 | 0.109 | 0.101 | 1.129 [1.080-1.177] | 0.25 | 0.95 |
| **rs780094** | *GCKR* | 2 | C/T | 0.573 | 0.618 | 0.6 | 0.929 [0.899-0.959] | 0.25 | 0.04 |
| **rs17791513** | *TLE4* | 9 | A/G | 0.949 | 0.952 | 0.939 | 1.168 [1.105-1.231] | 0.25 | 0.81 |
| **rs17168486** | *DGKB* | 7 | T/C | 0.181 | 0.172 | 0.163 | 1.100 [1.061-1.139] | 0.25 | 0.53 |
| **rs13233731** | *KLF14* | 7 | G/A | 0.551 | 0.538 | 0.527 | 1.077 [1.047-1.108] | 0.26 | 0.47 |
| **rs10886471** | *GRK5* | 10 | T/C | 0.47 | 0.477 | 0.489 | 0.932 [0.903-0.962] | 0.27 | 0.36 |
| **rs10830963** | *MTNR1B* | 11 | G/C | 0.282 | 0.27 | 0.29 | 0.932 [0.899-0.965] | 0.32 | 0.64 |
| **rs7955901** | *TSPAN8* | 12 | C/T | 0.464 | 0.487 | 0.438 | 1.061 [1.032-1.090] | 0.34 | 0.16 |
| **rs4275659** | *MPHOSPH9* | 12 | C/T | 0.715 | 0.735 | 0.694 | 1.065 [1.033-1.097] | 0.36 | 0.56 |
| **rs11063069** | *CCND2* | 12 | G/A | 0.214 | 0.223 | 0.221 | 0.932 [0.897-0.968] | 0.36 | 0.43 |
| **rs7041847** | *GLIS3* | 9 | A/G | 0.527 | 0.516 | 0.505 | 1.060 [1.029-1.090] | 0.37 | 0.40 |
| **rs7756992** | *CDKAL1* | 6 | G/A | 0.312 | 0.316 | 0.289 | 1.059 [1.027-1.091] | 0.40 | 0.79 |
| **rs9470794** | *ZFAND3* | 6 | T/C | 0.915 | 0.911 | 0.908 | 1.095 [1.044-1.147] | 0.41 | 0.65 |
| **rs1802295** | *VPS26A* | 10 | T/C | 0.304 | 0.321 | 0.322 | 0.944 [0.912-0.977] | 0.42 | 0.25 |
| **rs7403531** | *RASGRP1* | 15 | T/C | 0.218 | 0.229 | 0.204 | 1.064 [1.029-1.100] | 0.42 | 0.22 |
| **rs3802177** | *SLC30A8* | 8 | A/G | 0.31 | 0.294 | 0.301 | 1.055 [1.022-1.087] | 0.45 | 0.11 |
| **rs4812829** | *HNF4A* | 20 | G/A | 0.841 | 0.837 | 0.831 | 1.068 [1.027-1.109] | 0.45 | 0.37 |
| **rs243088** | *BCL11A* | 2 | T/A | 0.496 | 0.492 | 0.485 | 1.040 [1.010-1.070] | 0.54 | 0.41 |
| **rs12899811** | *PRC1* | 15 | G/A | 0.329 | 0.315 | 0.306 | 1.042 [1.010-1.073] | 0.55 | 0.87 |
| **rs12571751** | *ZMIZ1* | 10 | A/G | 0.564 | 0.583 | 0.549 | 1.040 [1.009-1.070] | 0.55 | 0.14 |
| **rs2334499** | *DUSP8* | 11 | T/C | 0.409 | 0.418 | 0.41 | 1.040 [1.009-1.070] | 0.55 | 0.30 |
| **rs7593730** | *RBMS1* | 2 | C/T | 0.793 | 0.811 | 0.796 | 1.048 [1.011-1.084] | 0.55 | 0.49 |
| **rs9936385** | *FTO* | 16 | C/T | 0.402 | 0.454 | 0.394 | 1.039 [1.008-1.069] | 0.56 | 1.18E-03 |
| **rs6795735** | *ADAMTS9* | 3 | C/T | 0.567 | 0.618 | 0.575 | 0.964 [0.934-0.993] | 0.56 | 0.01 |
| **rs10278336** | *GCK* | 7 | A/G | 0.586 | 0.577 | 0.579 | 1.038 [1.008-1.069] | 0.57 | 0.27 |
| **rs831571** | *PSMD6* | 3 | C/T | 0.835 | 0.81 | 0.827 | 1.047 [1.008-1.086] | 0.59 | 0.07 |
| **rs7202877** | *BCAR1* | 16 | T/G | 0.886 | 0.914 | 0.894 | 0.950 [0.902-0.997] | 0.61 | 0.04 |
| **rs7612463** | *UBE2E2* | 3 | C/A | 0.886 | 0.894 | 0.89 | 0.955 [0.907-1.003] | 0.65 | 0.17 |
| **rs4402960** | *IGF2BP* | 3 | T/G | 0.315 | 0.351 | 0.314 | 1.029 [0.997-1.062] | 0.68 | 0.05 |
| **rs702634** | *ARL15* | 5 | A/G | 0.711 | 0.711 | 0.701 | 1.027 [0.994-1.060] | 0.71 | 0.88 |
| **rs2028299** | *AP3S2* | 15 | C/A | 0.291 | 0.297 | 0.291 | 1.026 [0.993-1.059] | 0.72 | 0.69 |
| **rs2796441** | *TLE1* | 9 | G/A | 0.609 | 0.582 | 0.601 | 1.023 [0.992-1.053] | 0.73 | 0.14 |
| **rs11257655** | *CDC123* | 10 | T/C | 0.196 | 0.236 | 0.213 | 0.975 [0.937-1.012] | 0.75 | 0.12 |
| **rs6467136** | *GCC1* | 7 | A/G | 0.472 | 0.472 | 0.477 | 0.980 [0.950-1.010] | 0.75 | 0.60 |
| **rs1552224** | *ARAP1 (CENTD2)* | 11 | A/C | 0.848 | 0.849 | 0.85 | 0.975 [0.934-1.016] | 0.77 | 0.73 |
| **rs9505118** | *SSR1/RREB1* | 6 | A/G | 0.592 | 0.612 | 0.592 | 1.016 [0.986-1.047] | 0.81 | 0.17 |
| **rs7163757** | *C2CD4A* | 15 | C/T | 0.583 | 0.59 | 0.588 | 0.988 [0.958-1.018] | 0.85 | 0.15 |
| **rs7845219** | *TP53INP1* | 8 | T/C | 0.524 | 0.525 | 0.515 | 1.009 [0.980-1.039] | 0.88 | 0.45 |
| **rs6808574** | *LPP* | 3 | C/T | 0.62 | 0.604 | 0.613 | 1.009 [0.979-1.040] | 0.89 | 0.65 |
| **rs459193** | *ANKRD55* | 5 | G/A | 0.748 | 0.771 | 0.741 | 1.008 [0.974-1.043] | 0.91 | 0.13 |
| **rs6878122** | *ZBED3* | 5 | A/G | 0.706 | 0.658 | 0.706 | 0.995 [0.963-1.028] | 0.95 | 0.01 |
| **rs10842994** | *KLHDC5* | 12 | C/T | 0.797 | 0.814 | 0.794 | 1.004 [0.968-1.041] | 0.96 | 0.27 |
| **rs2075423** | *PROX1* | 1 | G/T | 0.66 | 0.678 | 0.653 | 0.999 [0.968-1.030] | 0.99 | 0.56 |

**Table S7: LADA cases positive for GADA and IA2A (n= 309) vs T2D (BMDCS controls)**

| **RSID** | **Locus** | **Chr** | **T2D Alleles Risk/Other** | **Risk Allele Frequency** | | | **LADA Odds Ratio** | **LADA *P*-value** | **LADA vs T2D *P*-value** |
| --- | --- | --- | --- | --- | --- | --- | --- | --- | --- |
|  |  |  |  | **LADA** | **T2D** | **Control** |  |  |  |
| **rs12427353** | *HNF1A* | 12 | G/C | 0.857 | 0.828 | 0.787 | 1.474 [1.438-1.511] | 2.52E-04 | 5.42E-02 |
| **rs8108269** | *GIPR* | 19 | G/T | 0.341 | 0.31 | 0.292 | 1.221 [1.189-1.253] | 3.25E-02 | 1.77E-01 |
| **rs6878122** | *ZBED3* | 5 | A/G | 0.744 | 0.658 | 0.706 | 1.216 [1.184-1.249] | 3.86E-02 | 1.47E-05 |
| **rs2943640** | *IRS1* | 2 | C/A | 0.674 | 0.675 | 0.634 | 1.209 [1.177-1.241] | 4.04E-02 | 9.59E-01 |
| **rs6723108** | *TMEM163* | 2 | T/G | 0.625 | 0.521 | 0.553 | 1.193 [1.164-1.223] | 4.20E-02 | 6.22E-03 |
| **rs7041847** | *GLIS3* | 9 | A/G | 0.554 | 0.516 | 0.505 | 1.188 [1.157-1.218] | 5.27E-02 | 1.68E-01 |
| **rs7903146** | *TCF7L2* | 10 | T/C | 0.251 | 0.376 | 0.298 | 0.852 [0.820-0.883] | 8.14E-02 | 2.56E-07 |
| **rs12571751** | *ZMIZ1* | 10 | A/G | 0.503 | 0.583 | 0.549 | 0.866 [0.836-0.895] | 9.35E-02 | 1.02E-03 |
| **rs2075423** | *PROX1* | 1 | G/T | 0.608 | 0.678 | 0.653 | 0.867 [0.837-0.898] | 1.11E-01 | 1.14E-02 |
| **rs7403531** | *RASGRP1* | 15 | T/C | 0.228 | 0.229 | 0.204 | 1.174 [1.138-1.209] | 1.22E-01 | 7.04E-01 |
| **rs849135** | *JAZF1* | 7 | G/A | 0.523 | 0.529 | 0.485 | 1.141 [1.112-1.171] | 1.30E-01 | 9.02E-01 |
| **rs17168486** | *DGKB* | 7 | T/C | 0.199 | 0.172 | 0.163 | 1.170 [1.131-1.208] | 1.63E-01 | 1.92E-01 |
| **rs4402960** | *IGF2BP* | 3 | T/G | 0.283 | 0.351 | 0.314 | 0.876 [0.843-0.908] | 1.65E-01 | 4.11E-03 |
| **rs7163757** | *C2CD4A* | 15 | C/T | 0.55 | 0.59 | 0.588 | 0.893 [0.864-0.922] | 1.87E-01 | 4.06E-02 |
| **rs11063069** | *CCND2* | 12 | G/A | 0.201 | 0.223 | 0.221 | 0.877 [0.841-0.912] | 1.98E-01 | 3.36E-01 |
| **rs459193** | *ANKRD55* | 5 | G/A | 0.764 | 0.771 | 0.741 | 1.133 [1.099-1.167] | 2.09E-01 | 6.51E-01 |
| **rs10842994** | *KLHDC5* | 12 | C/T | 0.816 | 0.814 | 0.794 | 1.141 [1.104-1.177] | 2.15E-01 | 8.39E-01 |
| **rs702634** | *ARL15* | 5 | A/G | 0.659 | 0.711 | 0.701 | 0.895 [0.863-0.927] | 2.31E-01 | 1.05E-01 |
| **rs163184** | *KCNQ1* | 11 | G/T | 0.497 | 0.49 | 0.463 | 1.104 [1.074-1.133] | 2.47E-01 | 5.68E-01 |
| **rs1802295** | *VPS26A* | 10 | T/C | 0.296 | 0.321 | 0.322 | 0.899 [0.867-0.932] | 2.59E-01 | 2.46E-01 |
| **rs9470794** | *ZFAND3* | 6 | T/C | 0.892 | 0.911 | 0.908 | 0.851 [0.801-0.900] | 2.64E-01 | 1.80E-01 |
| **rs17106184** | *FAF1* | 1 | G/A | 0.905 | 0.918 | 0.895 | 1.165 [1.117-1.213] | 2.73E-01 | 5.08E-01 |
| **rs3802177** | *SLC30A8* | 8 | G/A | 0.672 | 0.706 | 0.699 | 0.910 [0.878-0.942] | 3.13E-01 | 8.75E-02 |
| **rs7178572** | *HMG20A* | 15 | G/A | 0.707 | 0.73 | 0.73 | 0.910 [0.877-0.943] | 3.26E-01 | 2.48E-01 |
| **rs11257655** | *CDC123* | 10 | T/C | 0.189 | 0.236 | 0.213 | 0.900 [0.863-0.937] | 3.29E-01 | 1.22E-01 |
| **rs2028299** | *AP3S2* | 15 | C/A | 0.269 | 0.297 | 0.291 | 0.911 [0.878-0.944] | 3.34E-01 | 2.02E-01 |
| **rs4275659** | *MPHOSPH9* | 12 | C/T | 0.714 | 0.735 | 0.694 | 1.092 [1.060-1.124] | 3.49E-01 | 7.39E-01 |
| **rs10923931** | *NOTCH2* | 1 | T/G | 0.11 | 0.117 | 0.106 | 1.134 [1.088-1.181] | 3.52E-01 | 9.38E-01 |
| **rs780094** | *GCKR* | 2 | C/T | 0.566 | 0.618 | 0.6 | 0.923 [0.894-0.953] | 3.55E-01 | 1.77E-01 |
| **rs4458523** | *WFS1* | 4 | G/T | 0.574 | 0.614 | 0.59 | 0.930 [0.900-0.959] | 3.97E-01 | 1.20E-01 |
| **rs1359790** | *SPRY2* | 13 | G/A | 0.717 | 0.741 | 0.736 | 0.925 [0.892-0.958] | 4.21E-01 | 8.02E-02 |
| **rs7845219** | *TP53INP1* | 8 | T/C | 0.495 | 0.525 | 0.515 | 0.933 [0.903-0.963] | 4.24E-01 | 3.70E-01 |
| **rs10886471** | *GRK5* | 10 | T/C | 0.468 | 0.477 | 0.489 | 0.935 [0.906-0.965] | 4.32E-01 | 5.75E-01 |
| **rs3786897** | *PEPD* | 19 | A/G | 0.57 | 0.578 | 0.589 | 0.935 [0.905-0.965] | 4.47E-01 | 5.84E-01 |
| **rs16861329** | *ST64GAL1* | 3 | C/T | 0.872 | 0.872 | 0.861 | 1.097 [1.055-1.140] | 4.50E-01 | 6.59E-01 |
| **rs10830963** | *MTNR1B* | 11 | G/C | 0.314 | 0.27 | 0.29 | 1.070 [1.037-1.103] | 4.77E-01 | 4.90E-02 |
| **rs5215** | *KCNJ11* | 11 | C/T | 0.374 | 0.382 | 0.362 | 1.065 [1.034-1.095] | 4.85E-01 | 9.76E-01 |
| **rs11634397** | *ZFAND6* | 15 | G/A | 0.668 | 0.679 | 0.653 | 1.062 [1.032-1.093] | 4.97E-01 | 4.43E-01 |
| **rs10401969** | *CILP2* | 19 | C/T | 0.06 | 0.088 | 0.067 | 0.888 [0.828-0.948] | 4.99E-01 | 2.40E-02 |
| **rs4430796** | *HNF1B* | 17 | G/A | 0.5 | 0.479 | 0.484 | 1.060 [1.029-1.090] | 5.09E-01 | 3.44E-01 |
| **rs6467136** | *GCC1* | 7 | A/G | 0.492 | 0.472 | 0.477 | 1.054 [1.024-1.083] | 5.41E-01 | 8.66E-01 |
| **rs2261181** | *HMGA2* | 12 | T/C | 0.115 | 0.109 | 0.101 | 1.084 [1.036-1.133] | 5.69E-01 | 5.69E-01 |
| **rs10203174** | *THADA* | 2 | C/T | 0.879 | 0.897 | 0.871 | 1.065 [1.021-1.109] | 6.21E-01 | 1.05E-01 |
| **rs11717195** | *ADCY5* | 3 | T/C | 0.757 | 0.777 | 0.77 | 0.951 [0.915-0.987] | 6.29E-01 | 6.77E-01 |
| **rs1111875** | *HHEX/IDE* | 10 | C/T | 0.587 | 0.634 | 0.59 | 0.960 [0.931-0.990] | 6.35E-01 | 9.02E-03 |
| **rs243088** | *BCL11A* | 2 | T/A | 0.489 | 0.492 | 0.485 | 1.041 [1.011-1.070] | 6.41E-01 | 4.54E-01 |
| **rs6813195** | *TMEM154* | 4 | C/T | 0.723 | 0.737 | 0.718 | 1.044 [1.011-1.077] | 6.52E-01 | 8.01E-01 |
| **rs516946** | *ANK1* | 8 | C/T | 0.751 | 0.782 | 0.754 | 1.037 [1.004-1.071] | 7.08E-01 | 9.86E-02 |
| **rs10278336** | *GCK* | 7 | A/G | 0.567 | 0.577 | 0.579 | 0.968 [0.937-0.998] | 7.09E-01 | 6.17E-01 |
| **rs3923113** | *GRB14* | 2 | A/C | 0.639 | 0.656 | 0.632 | 1.033 [1.003-1.064] | 7.14E-01 | 2.16E-01 |
| **rs391300** | *SRR* | 17 | C/T | 0.618 | 0.638 | 0.61 | 1.032 [1.002-1.061] | 7.20E-01 | 5.31E-01 |
| **rs7593730** | *RBMS1* | 2 | C/T | 0.794 | 0.811 | 0.796 | 1.039 [1.002-1.075] | 7.23E-01 | 4.01E-01 |
| **rs7612463** | *UBE2E2* | 3 | C/A | 0.887 | 0.894 | 0.89 | 0.953 [0.904-1.001] | 7.31E-01 | 3.48E-01 |
| **rs7955901** | *TSPAN8* | 12 | C/T | 0.456 | 0.487 | 0.438 | 1.028 [0.999-1.057] | 7.47E-01 | 8.39E-02 |
| **rs12970134** | *MC4R* | 18 | A/G | 0.257 | 0.291 | 0.247 | 1.032 [0.998-1.066] | 7.50E-01 | 1.88E-01 |
| **rs6795735** | *ADAMTS9* | 3 | C/T | 0.579 | 0.618 | 0.575 | 1.027 [0.997-1.056] | 7.57E-01 | 2.46E-01 |
| **rs4812829** | *HNF4A* | 20 | G/A | 0.826 | 0.837 | 0.831 | 0.970 [0.931-1.010] | 7.97E-01 | 5.08E-01 |
| **rs831571** | *PSMD6* | 3 | C/T | 0.817 | 0.81 | 0.827 | 0.974 [0.935-1.012] | 8.13E-01 | 9.28E-01 |
| **rs1552224** | *ARAP1 (CENTD2)* | 11 | A/C | 0.859 | 0.849 | 0.85 | 1.025 [0.983-1.066] | 8.40E-01 | 3.23E-01 |
| **rs17584499** | *PTPRD* | 9 | T/C | 0.202 | 0.17 | 0.193 | 1.020 [0.984-1.056] | 8.49E-01 | 1.43E-01 |
| **rs2796441** | *TLE1* | 9 | G/A | 0.602 | 0.582 | 0.601 | 1.015 [0.986-1.045] | 8.61E-01 | 2.25E-01 |
| **rs9505118** | *SSR1/RREB1* | 6 | A/G | 0.602 | 0.612 | 0.592 | 1.015 [0.985-1.045] | 8.65E-01 | 6.60E-01 |
| **rs6808574** | *LPP* | 3 | C/T | 0.623 | 0.604 | 0.613 | 1.009 [0.980-1.039] | 9.14E-01 | 8.13E-01 |
| **rs10811661** | *CDKN2A/B* | 9 | T/C | 0.832 | 0.858 | 0.831 | 1.011 [0.972-1.049] | 9.26E-01 | 3.70E-01 |
| **rs9936385** | *FTO* | 16 | C/T | 0.396 | 0.454 | 0.394 | 1.008 [0.978-1.037] | 9.30E-01 | 2.42E-02 |
| **rs12899811** | *PRC1* | 15 | G/A | 0.314 | 0.315 | 0.306 | 0.995 [0.964-1.026] | 9.56E-01 | 9.26E-01 |
| **rs13233731** | *KLF14* | 7 | G/A | 0.529 | 0.538 | 0.527 | 1.004 [0.974-1.034] | 9.66E-01 | 2.41E-01 |
| **rs2334499** | *DUSP8* | 11 | T/C | 0.409 | 0.418 | 0.41 | 0.997 [0.967-1.027] | 9.75E-01 | 2.40E-01 |
| **rs7756992** | *CDKAL1* | 6 | G/A | 0.294 | 0.316 | 0.289 | 1.001 [0.969-1.033] | 9.89E-01 | 7.67E-01 |
| **rs7202877** | *BCAR1* | 16 | T/G | 0.895 | 0.914 | 0.894 | 0.999 [0.952-1.046] | 9.94E-01 | 2.00E-01 |
| **rs17791513** | *TLE4* | 9 | A/G | 0.939 | 0.952 | 0.939 | 1.001 [0.941-1.062] | 9.94E-01 | 3.10E-01 |

**Supplementary Results**

Given our primary control cohort consisted of European-descent children ascertained from the United States, while the LADA cases were adults ascertained from the UK and Germany, we also leveraged 2,820 healthy adult British controls from the WTCCC and, overall, observed consistent results (**Supplemental Table 8**), despite the array differences for this control set, as outlined in the Methods section. The T1D signals at the MHC (OR=1.18, *P*=1.01x10^-4^), *PTPN22* (OR=1.33; *P*=3.86x10^-6^), *SH2B3* (OR=1.16; *P*=9.35x10^-5^), and *INS* (rs689; OR=1.24; *P*=1.27x10^-6^) remained strongly associated and were directionally consistent. Similar observations were also seen when comparing the MHC (*P*_difference_=1.26x10^-17^), *INS* (rs689; *P*_difference_= 3.88x10^-4^), and *SMARCE1* (*P*_difference_= 6.54x10^-4^) loci in the LADA cases vs the WTCCC T1D cases. While the *HNF1A* association weakened, it did remain significantly associated (OR=1.11; *P*=0.036) while the *TCF7L2* signal remained significantly differently associated in LADA cases vs T1D cases (*P*_difference_=5.21x10*^-^*^6^).

When constrained on GADA+ only LADA cases, the signal at the MHC was no longer significantly associated while *TCF7L2* remined non-significant for cases vs WTCCC controls; however they both remained significantly different between LADA cases vs T1D cases (*P*_difference_=1.99x10^-24^ and 5.03x10^-4^, respectively). Furthermore, when constrained on LADA cases positive for GADA+ and IA2A+ antibodies, the T1D association signals remained all significant, although not all were significantly different from T1D, i.e. MHC (OR=1.59, *P*=3.34x10^-9^, *P*_difference_=4.01x10^-3^), *PTPN22* (OR=1.86; *P*=1.88x10^-7^, *P*_difference_= N.S), *SH2B3* (OR=1.32; *P*=1.95x10^-4^, *P*_difference_= N.S), and *INS* (rs689; OR=1.48; *P*=2.07x10^-6^ , *P*_difference_=1.68x10^-6^) showing again that this set of LADA cases is overall more similar to T1D cases. The *HNF1A* signal remained significantly associated in these restricted LADA cases (OR=1.27; *P*=0.011), while the *ZBED3* and *TCF7L2* loci continued to be significantly different in LADA cases vs T1D cases (*P*_difference_=1.47x10^-5^ and 2.56x10^-7^, respectively).

**Table S8: Follow-up analysis of significant T1D and T2D loci in LADA using WTCCC controls**

A) MHC, *PTPN22, SH2B3, INS, SMARCE1* association replicated in 978 LADA cases and 2,820 WTCCC healthy British controls

| **Locus** | **SNP** | **T1D Alleles Risk/Other** | **Risk Allele Frequency** | | | **LADA Odds Ratio** | **LADA *P*-value** | **LADA vs T1D *P*-value** |
| --- | --- | --- | --- | --- | --- | --- | --- | --- |
|  |  |  | **LADA** | **T1D** | **Control** |  |  |  |
| *MHC* | rs9272346 | A/G | 0.686 | 0.818 | 0.620 | 1.177[1.161-1.192] | 1.01x10^-4^ | 1.26x10^-17^ |
| *PTPN22* | rs6679677 | A/C | 0.143 | 0.17 | 0.097 | 1.329[1.306-1.352] | 3.86x10^-6^ | 2.61x10^-2^ |
| *SH2B3* | rs17696736 | G/A | 0.515 | 0.503 | 0.44 | 1.162[1.148-1.177] | 9.35 x10^-5^ | 0.542 |
| *INS* | rs689 | T/A | 0.796 | 0.741 | 0.712 | 1.235[1.219-1.252] | 1.27x10^-4^ | 3.88x10^-4^ |
| *SMARCE1* | rs7221109 | C/T | 0.621 | 0.687 | 0.648 | 1.010[0.995-1.025] | 0.810 | 6.54x10^-4^ |

B) MHC, *PTPN22, SH2B3, and INS* association replicated in 309 LADA cases positive for autoantibodies GADA and IA2 and 2,820 WTCCC healthy British controls

| **Locus** | **SNP** | **T1D Alleles Risk/Other** | **Risk Allele Frequency** | | | **LADA Odds Ratio** | **LADA *P*-value** | **LADA vs T1D *P*-value** |
| --- | --- | --- | --- | --- | --- | --- | --- | --- |
|  |  |  | **LADA** | **T1D** | **Control** |  |  |  |
| *MHC* | rs9272346 | A/G | 0.763 | 0.818 | 0.620 | 1.591 [1.577-1.604] | 3.34x10^-9^ | 4.01x10^-3^ |
| *PTPN22* | rs6679677 | A/C | 0.17 | 0.17 | 0.097 | 1.855 [1.834-1.875] | 1.88x10^-7^ | 0.603 |
| *SH2B3* | rs17696736 | G/A | 0.542 | 0.503 | 0.44 | 1.316 [1.303-1.329] | 1.95x10^-4^ | 0.180 |
| *INS* | rs689 | T/A | 0.824 | 0.741 | 0.712 | 1.479 [1.465-1.494] | 2.07x10^-6^ | 1.68x10^-6^ |
| *INS* | rs7111341 | C/T | 0.812 | 0.75 | 0.734 | 1.272 [1.258-1.287] | 4.05x10^-3^ | 2.39x10^-4^ |

C) *HNF1A* and *TCF7L2* association replicated in 978 LADA cases and 2,820 WTCCC healthy British controls

| **Locus** | **SNP** | **T2D Alleles Risk/Other** | **Risk Allele Frequency** | | | **LADA Odds Ratio** | **LADA *P*-value** | **LADA vs T2D *P*-value** |
| --- | --- | --- | --- | --- | --- | --- | --- | --- |
|  |  |  | **LADA** | **T2D** | **Control** |  |  |  |
| *HNF1A* | rs12427353 | G/C | 0.831 | 0.828 | 0.807 | 1.111 [1.092-1.129] | 3.57x10^-2^ | 0.538 |
| *TCF7L2* | rs7903146 | T/C | 0.295 | 0.376 | 0.300 | 0.988 [0.972-1.004] | 0.702 | 5.21x10^-6^ |

D) *HNF1A, ZBED3, and TCF7L2* association replicated in 309 LADA cases positive for autoantibodies GADA and IA2 and 2,820 WTCCC healthy British controls

| **Locus** | **SNP** | **T2D Alleles Risk/Other** | **Risk Allele Frequency** | | | **LADA Odds Ratio** | **LADA *P*-value** | **LADA vs T2D *P*-value** |
| --- | --- | --- | --- | --- | --- | --- | --- | --- |
|  |  |  | **LADA** | **T2D** | **Control** |  |  |  |
| *HNF1A* | rs12427353 | G/C | 0.857 | 0.828 | 0.807 | 1.274 [1.258-1.290] | 1.05x10^-2^ | 5.42x10^-2^ |
| *ZBED3* | rs6878122 | A/G | 0.744 | 0.658 | 0.658 | 1.132 [1.118-1.146] | 0.123 | 1.47x10^-5^ |
| *TCF7L2* | rs7903146 | T/C | 0.251 | 0.376 | 0.300 | 0.851 [0.837-0.865] | 4.62x10^-2^ | 2.56x10^-7^ |

**Figure S1: Principle Component Analyses.** A) 978 LADA cases (red) vs 1,057 BMDCS controls (blue), and B) 978 LADA cases (red) vs 2,820 WTCCC controls (blue).


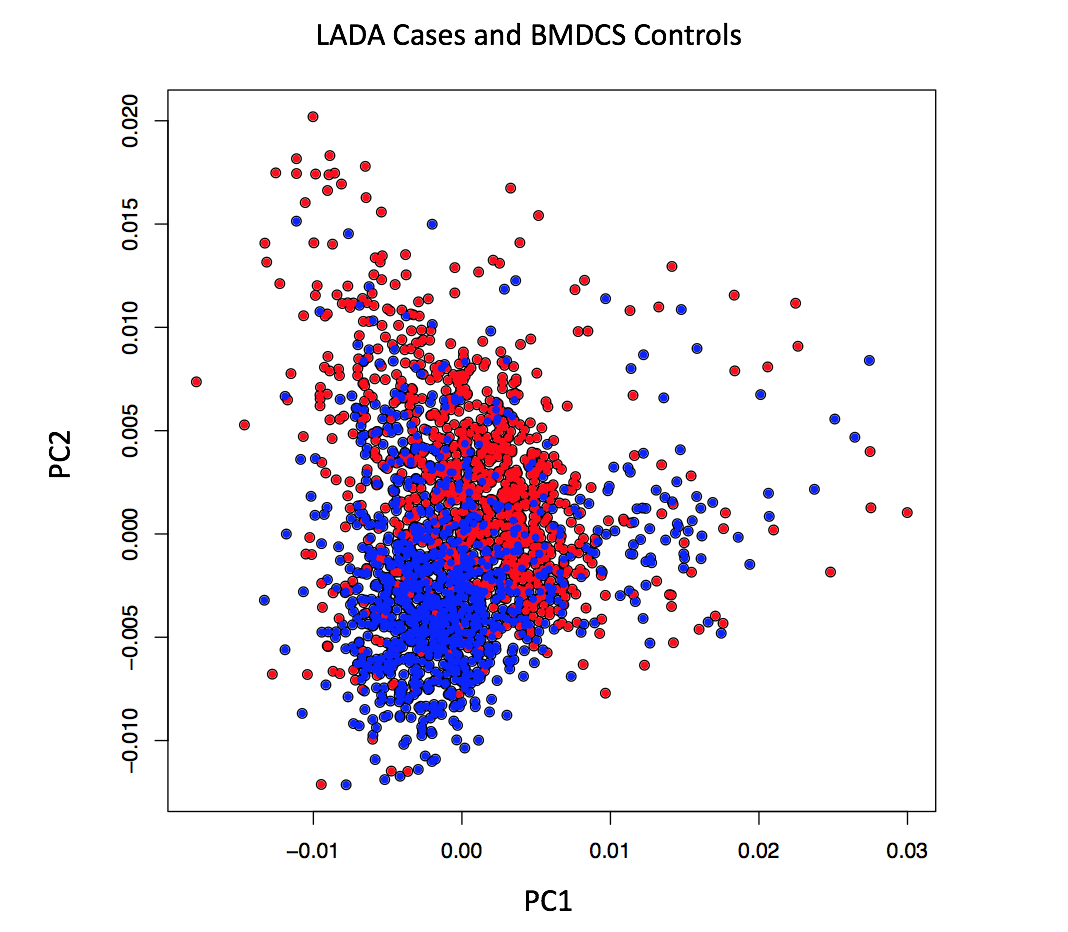


**A**

**B**


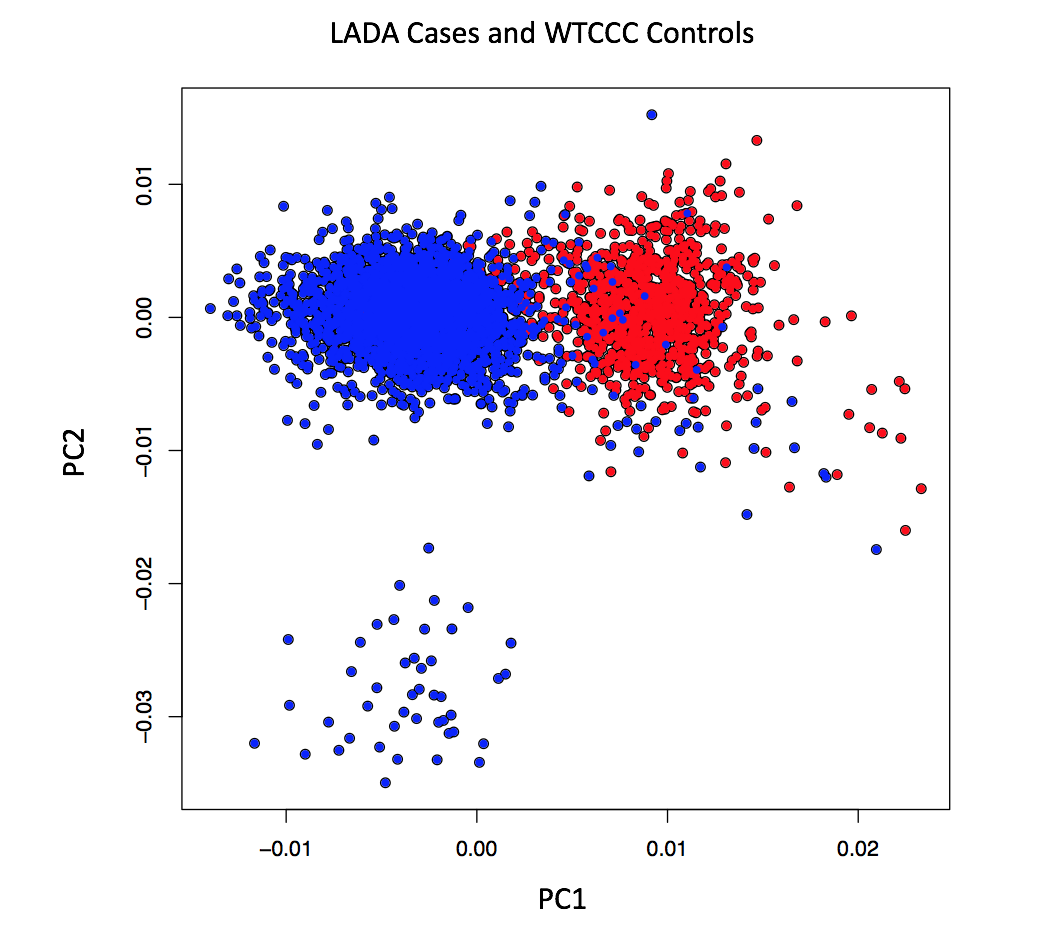


**Figure S2**

Quantile-quantile plot of pruned markers used for PCs (no HLA signals) for LADA cases and BMDCS controls. The genomic inflation factor is 0.966.

**
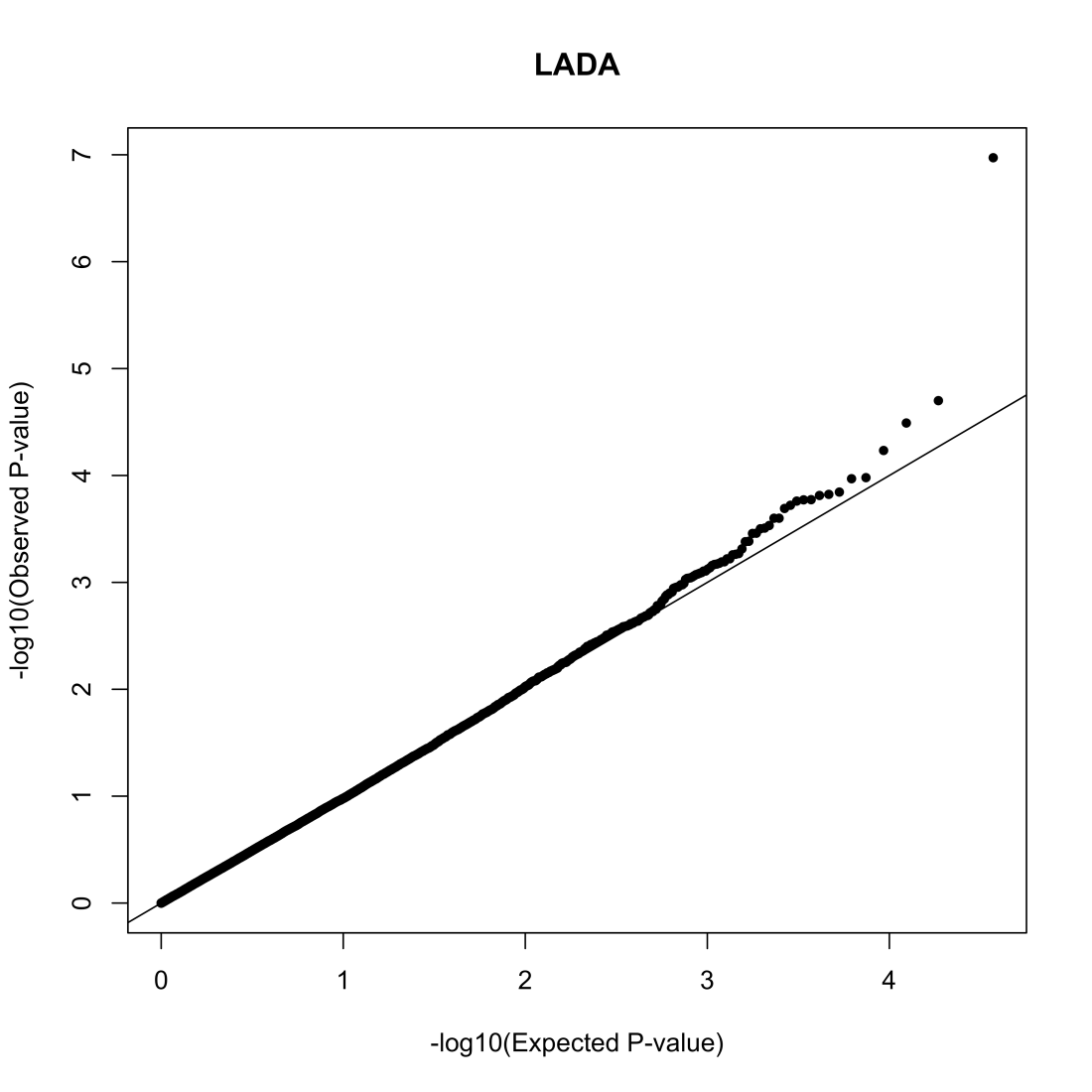
**

**Figure S3**

**Extended set of ROC plots including Non-HLA and HLA only GRS also tested in LADA vs T1D and GADA+ only LADA vs GADA+IA2A LADA.** Weighted genetic risk scores (GRS) for T1D GRS (black), T1D without HLA GRS (blue) , HLA only GRS (cyan), T2D GRS (red) and both T1D and T2D GRS (green) were calculated and tested in A) 978 LADA cases and 1,057 healthy controls B) 669 GADA only autoantibody- positive and 1,057 healthy controls C) 309 autoantibody-positive (GADA, IA2A) LADA and 1,057 controls D) 978 LADA and 1,990 WTCCC T1D cases E) 669 GADA only autoantibody- positive LADA and 1,990 WTCCC T1D F) 309 autoantibody- positive (GADA, IA2A) LADA and 1,990 WTCCC T1D. The T2D GRS predict GADA+ only LADA from GADA+IA2A+ LADA and LADA from T1D, resulting in T2D GRS performance dropping below the y=x line.


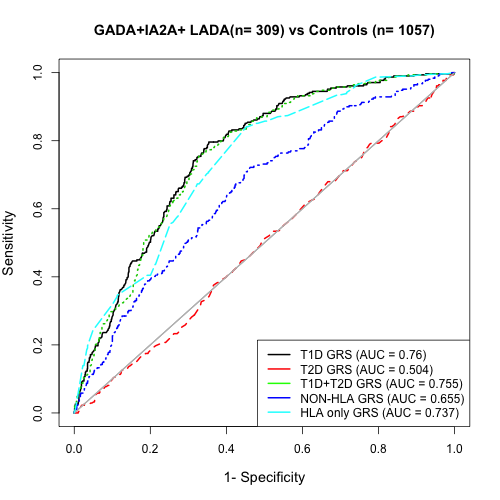

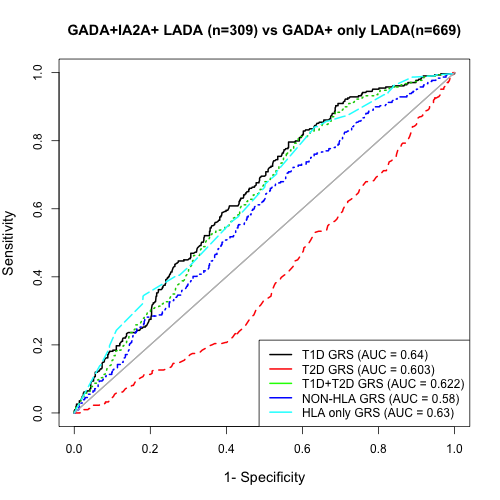

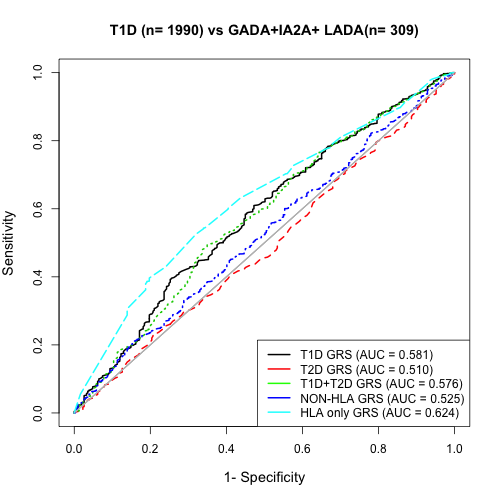

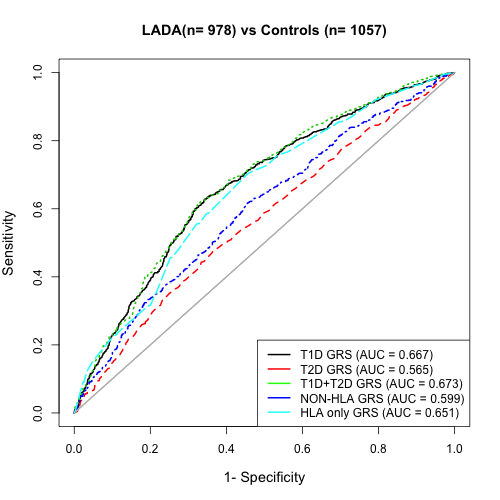

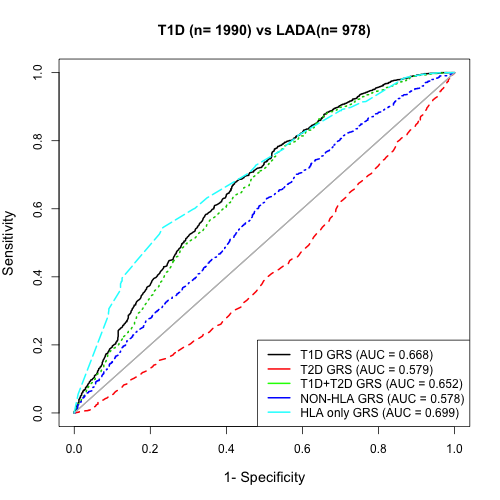

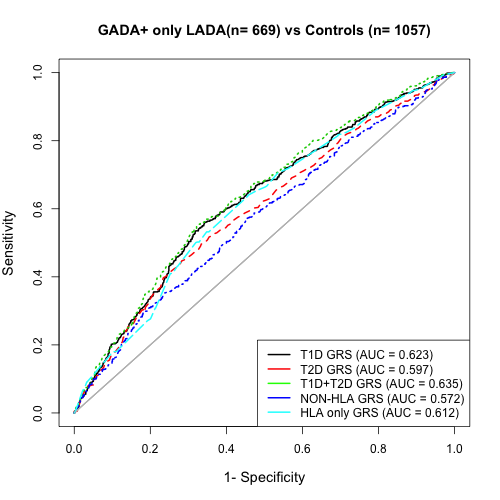

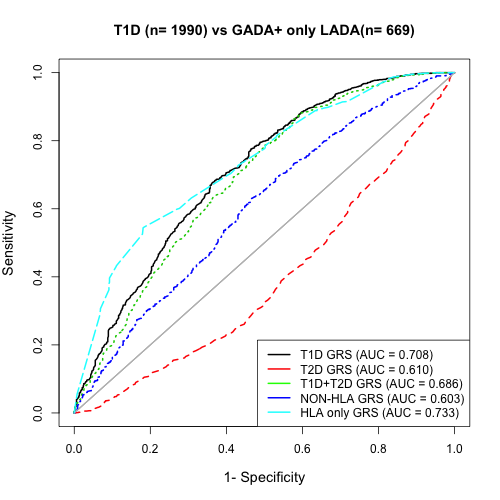


A

B

C

D

E

F

G

**Supplemental Methods**

Study populations and antibody testing – full description

We ascertained 978 LADA cases from two studies, a European Union-funded multicenter study (Action LADA) and a German Research Council study (DFG: SFB 518, A1), each of which aims to identify features of adult-onset autoimmune diabetes. A description of the participants and the study design has been published elsewhere^1^. Briefly, for this study, all participants were diagnosed with LADA if: aged 30-70 years old, positive for diabetes-associated Glutamic Acid Decarboxylase (GAD) autoantibodies, without insulin treatment for at least 6 months after diagnosis. These cross-sectional studies included adult-onset diabetic patients recruited between 2003 and 2007 from Barcelona (Spain), Düsseldorf (Germany), London (UK), Odense (Denmark) and Ulm (Germany); two centers recruited patients from a community or primary care setting (Düsseldorf and Odense), and the remaining three centers (Barcelona, London and Ulm) recruited patients in a hospital setting. The ethics committees of all participating centers approved the protocol, and all participants provided written informed consent. Patients were diagnosed with diabetes according to standard criteria, with at least two recorded fasting blood glucose measurements ≥7mmol/L, or 2 hour post-oral glucose blood glucose >10mM. Patients were excluded if their data was incomplete, they were pregnant, had renal disease (raised creatinine or proteinuria) or an acute illness at the time of testing. An attending physician recorded the medication data and risk factors. Serum and plasma samples were collected according to standard procedures and stored at -20°C.

Samples were tested in two central laboratories (London, UK and Ulm, Germany) for serum autoantibodies to glutamic acid decarboxylase (GADA) and insulinoma associated antigen-2 (IA2A), using established radioimmunoprecipitation assays. Positive results were replicated, reducing false positives to <0·2%. Using the Diabetes Antibody Standardization Program^2^ (and unpublished data), we determined the sensitivity and specificity of the GADA assays (90%, 93% in London and 86%, 95% in Ulm, respectively) and the sensitivity and specificity of IA2A assays (68%, 95% in London and 73%, 99% in Ulm, respectively)^2^.

Each assay included serially diluted sera from antibody positive individuals. The cut-off for positivity was selected arbitrarily based on the end point of the standard curve and further confirmed with Quantile-Quantile probability plots for London (UK) and the 99^th^ centile for Ulm (Germany).

The population-based control cohort comprised non-diabetic children of European ancestry, aged 5-20 years old, enrolled in the Bone Mineral Density in Childhood Study (BMDCS). Subjects were randomly recruited from five different centers in the US. As previously reported^3^, enrollment criteria included healthy, normally developing children. Each participating center received approval of the study by their respective institutional review boards. Participants 18 years old and older provided written informed consent. Written informed consent for participants younger than 18 years of age was obtained from the parent or guardian and assent was obtained from the participants.

**Genotyping**

LADA samples and BMDCS controls were genotyped on the Illumina Infinium II OMNI Express plus Exome BeadChip array (Illuminia, San Diego, CA, USA) at the Children’s Hospital of Philadelphia Center for Applied Genomics (Philadelphia, PA, USA). Our study also utilized publicly available childhood-onset T1D (n=2,000) and adult-onset T2D (n=1,999) Affymetrix 500K genotype data from the Wellcome Trust Case Control Consortium; these individuals were recruited within England, Scotland and Wales^4^. WTCCC control samples consisted of 3,000 1958 British Birth Cohort control samples genotyped on the Illumina 660K genotyping array. Individual data from WTCCC is available through the Consortium’s Data access committee (http://www.wtccc.org.uk).

**Quality Control and Imputation**

PLINK^5^ was used to exclude individuals with incorrect gender assignments or whose gender could not be determined by genotype, duplicate individuals, and individuals with missing genotype rate >5%. Principal component analysis was used to exclude ethnic outliers. Single nucleotide polymorphisms (SNPs) with a call rate <95%, minor allele frequency <0.5%, Hardy-Weinberg equilibrium *P*<10^-5^ and with A/T and G/C alleles were removed. After quality control, genotypes were imputed to the 1000 Genome Phase I Integrated Release Version 3 reference panel. A two-step imputation process was performed using SHAPEIT^6^ for haplotype phasing and IMPUTE2^7^ for imputation. All SNPs in this study had imputation quality scores >0.40.

1. Hawa MI, Kolb H, Schloot N, et al. Adult-onset autoimmune diabetes in Europe is prevalent with a broad clinical phenotype: Action LADA 7. *Diabetes care.* 2013;36(4):908-913.

2. Torn C, Mueller PW, Schlosser M, Bonifacio E, Bingley PJ, Participating L. Diabetes Antibody Standardization Program: evaluation of assays for autoantibodies to glutamic acid decarboxylase and islet antigen-2. *Diabetologia.* 2008;51(5):846-852.

3. Kalkwarf HJ, Zemel BS, Gilsanz V, et al. The bone mineral density in childhood study: bone mineral content and density according to age, sex, and race. *The Journal of clinical endocrinology and metabolism.* 2007;92(6):2087-2099.

4. Wellcome Trust Case Control C. Genome-wide association study of 14,000 cases of seven common diseases and 3,000 shared controls. *Nature.* 2007;447(7145):661-678.

5. Purcell S, Neale B, Todd-Brown K, et al. PLINK: a tool set for whole-genome association and population-based linkage analyses. *Am J Hum Genet.* 2007;81(3):559-575.

6. O'Connell J, Gurdasani D, Delaneau O, et al. A general approach for haplotype phasing across the full spectrum of relatedness. *PLoS genetics.* 2014;10(4):e1004234.

7. Howie B, Fuchsberger C, Stephens M, Marchini J, Abecasis GR. Fast and accurate genotype imputation in genome-wide association studies through pre-phasing. *Nature genetics.* 2012;44(8):955-959.
